# Hydroxyethylcellulose- and Chitosan-Modified 3D-Printed Carbon Electrodes for Indole-3-Acetic Acid and Salicylic Acid Determination

**DOI:** 10.1021/acsomega.6c07701

**Published:** 2026-09-21

**Authors:** Pablo H. S. Martins, Hugo L. S. L. Leão, Polyana F. Pereira

**Affiliations:** Instituto Federal Goiano, Campus Rio Verde - Rod. Sul Goiana Km 01, Zona Rural, Rio Verde, Goiás 75901-970, Brazil

## Abstract

This study describes the development of a 3D-printed carbon black (3D-CB) electrode modified with hydroxyethylcellulose (HEC) and chitosan (CH) layers, applied to the simultaneous quantification of indole-3-acetic acid (IAA) and salicylic acid (SA) in plant samples. Measurements were performed by square-wave voltammetry (SWV) under optimized conditions, enabling rapid and resolved detection of phytohormones. The method exhibited linear responses for both analytes, with correlation coefficients (r) of 0.999. Limits of detection (LOD) were 0.446 μM for IAA and 0.335 μM for SA. Good precision was obtained, with RSD values for both intraday and interday analyses not exceeding 4.70%. The applicability of the method was evaluated in real plant samples, including roots and leaves of soybean and maize, as well as roots, leaves, and green fruits of strawberry. For IAA, mean recoveries for the three extracts ranged from 96.82% to 101.37%, with RSD values between 2.74% and 5.27%. For SA, recoveries ranged from 98.13% to 102.98%, with RSD values between 1.92% and 4.01%. The absence of a statistically significant difference between added and found concentrations confirmed the accuracy and reliability of the proposed method. Owing to its simple fabrication, rapid SWV response, low operational cost, and use of environmentally friendly materials, the proposed 3D-CB/HEC–CH platform represents an alternative for efficient monitoring of IAA and SA in agricultural matrices.

## Graphical abstract

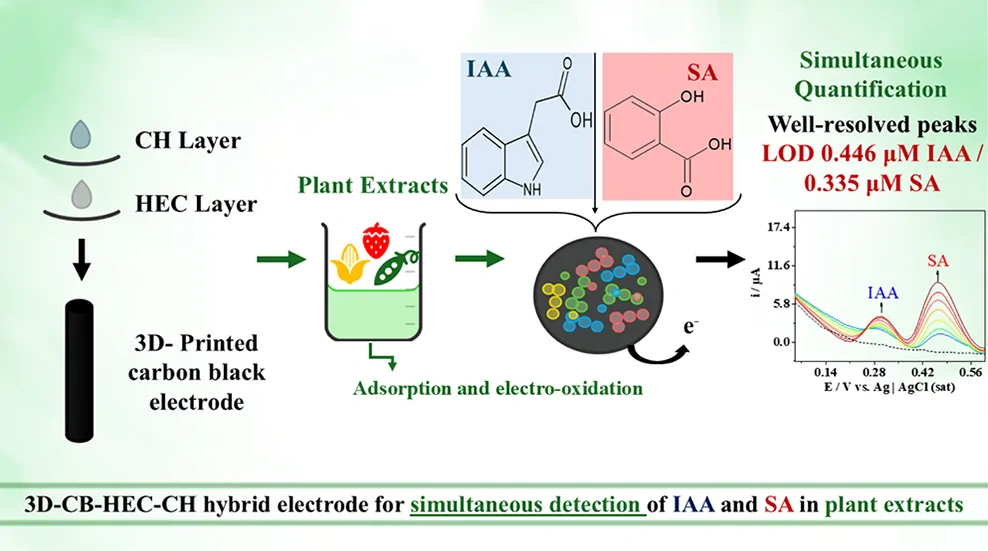

## 1 Introduction

Plant physiological processes are regulated by organic molecules known as phytohormones, with indole-3-acetic acid (IAA) and salicylic acid (SA) standing out as important regulators due to their central, and often antagonistic, roles in coordinating plant growth and defense responses.^1^ IAA mainly regulates growth and developmental processes, including cell elongation, organogenesis, and root–shoot morphogenesis.^2^ Beyond these classical roles, recent studies have revealed that IAA contributes to plant adaptation to abiotic stresses such as salinity,^3^ drought,^4^ and nutrient deficiency.^5^ SA is an indispensable molecule that modulates growth and development by enhancing photosynthetic efficiency, activating antioxidant defenses, and promoting osmoprotectant accumulation.^6^

While IAA and SA are commonly studied separately, recent studies have shown that both act in a coordinated manner in plant stress responses, with SA signaling directly modulating auxin transport and distribution. This interaction between these phytohormones reinforces the need for analytical approaches capable of simultaneously determining IAA and SA under complex physiological conditions.^7^ Although chromatographic techniques dominate the literature on simultaneous IAA and SA determination,^8–10^ nonchromatographic methods such as colorimetric,^11^ spectrofluorimetric,^12^ and immunoassays^13,14^ are predominantly employed for isolated quantification. However, challenges regarding selectivity, sensitivity, and matrix effects limit their applicability for reliable simultaneous analysis in complex plant matrices.^12^ An overview of the physiological roles of IAA and SA, current analytical techniques, their limitations, and emerging trends in electrochemical sensing is presented in Figure 1.

**Figure 1:**
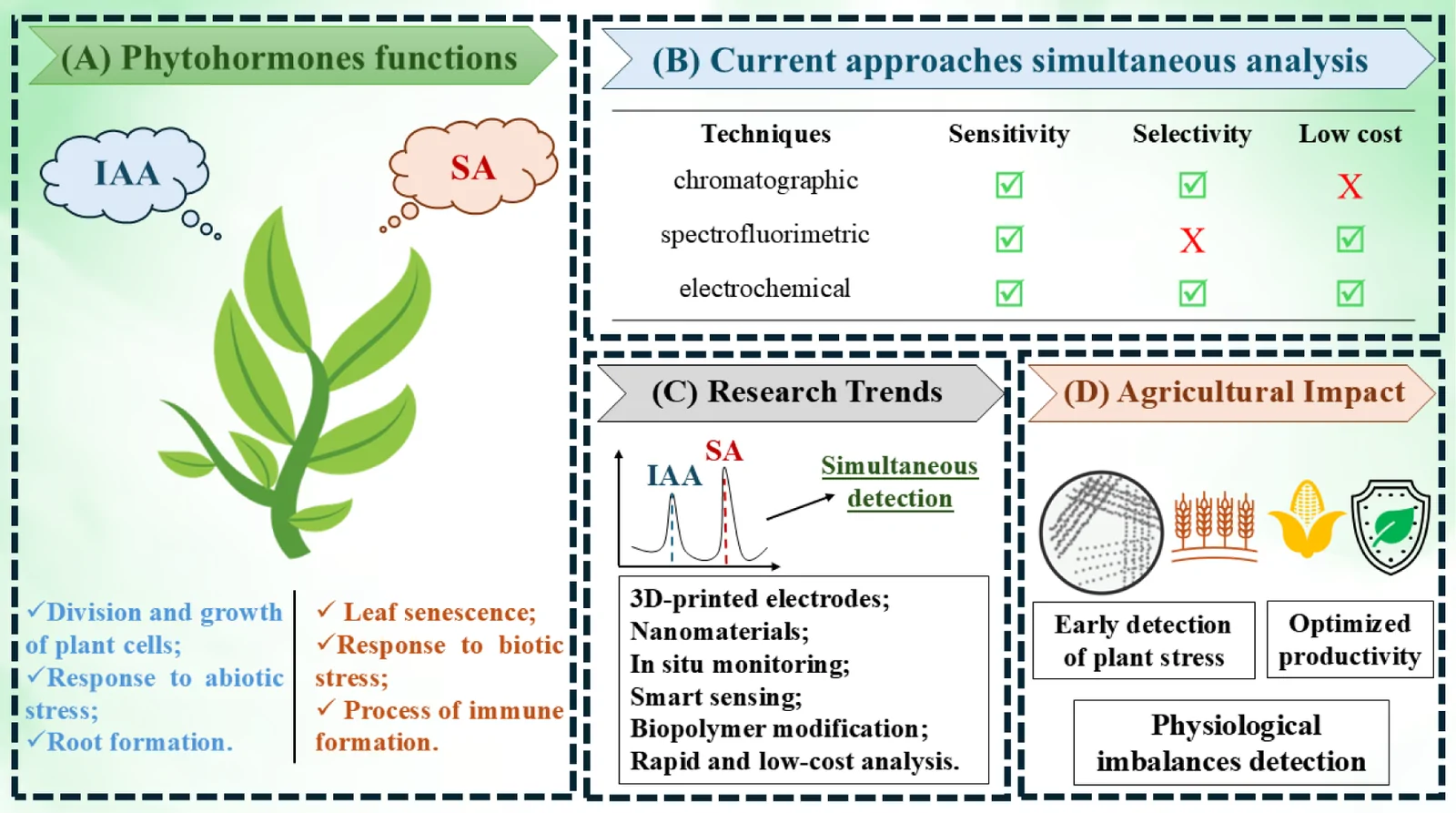
Overview of the physiological roles of IAA and SA in plants and current approaches for their simultaneous analysis: (a) physiological functions of IAA and SA in plants; (b) comparison of current analytical techniques in terms of sensitivity, selectivity, and cost; (c) emerging research trends for simultaneous IAA and SA detection, highlighting 3D-printed electrodes, nanomaterials, in situ monitoring, smart sensing, biopolymer modification, and rapid, low-cost analysis; and (d) potential agricultural applications, including early detection of plant stress, identification of physiological imbalances, and optimization of crop productivity.

Voltammetric techniques offer a compelling alternative for IAA and SA determination due to their operational simplicity, strong analytical performance, rapid response, and ease of implementation.^15–17^ Several limitations remain in the current literature. The simultaneous detection of IAA and SA requires correction procedures for voltammetric signals to resolve overlapping signals. The electrochemical response is influenced by analyte diffusion, which compromises measurement accuracy and reproducibility. Certain electrodes exhibited variations of 10–13%, attributed to reproducibility limitations. These challenges highlight the need for electrochemical platforms with enhanced selectivity, stability, and analytical performance.^18,19^

In recent years, particular attention has been given to carbon-based electrodes, especially those with three-dimensional (3D) architectures, which have emerged as frontrunners for enabling low-cost and customizable platforms.^20,21^ The use of biopolymers such as chitosan (CH) has further enhanced the applicability of these 3D platforms, providing analyte affinity while maintaining compatibility with advanced voltammetric techniques for phytohormone analysis.^17,22,23^

The use of CH as the sole matrix material is associated with inherent limitations, including film swelling, pH-dependent solubility, limited mechanical stability, and difficulties in achieving homogeneous and reproducible coatings in aqueous and complex sample conditions.^24,25^ Nanoparticles and conductive materials have been incorporated into CH films to improve strength, conductivity, and stability.^26^ Another approach is the addition of hydroxyethylcellulose (HEC). Previous work by these authors has shown that CH films with HEC are more uniform and more resistant, and they produce more stable signals,^27^ thereby supporting more reliable electrochemical detection of phytohormones such as IAA and SA.

In this work, a novel method comprising a 3D-printed carbon black (3D-CB) electrode functionalized with a hydroxyethylcellulose/chitosan (HEC–CH) bilayer for the rapid and simultaneous electrochemical determination of IAA and SA by square-wave voltammetry (SWV) was proposed. Although progress has been made in electrochemical sensors for IAA and SA determination, most of the previously reported approaches involve multistep fabrication protocols and elaborate surface modifications, which raise production costs. 3D printing, by contrast, offers a rapid, low-cost, and customizable route to electrode fabrication, and the HEC–CH bilayer can be deposited through a straightforward modification step that eliminates the need for sophisticated functionalization or nanostructured materials, yielding a simple, reproducible, cost-effective, and environmentally friendly platform for phytohormone analysis. No previous studies have described the integration of 3D-printed carbon electrodes with cellulose-derived biopolymers for the simultaneous voltammetric detection of these phytohormones. The applicability was demonstrated through the analysis of soybean (*Glycine max*), maize (*Zea mays*) and strawberry (*Fragaria × ananassa*) extracts, highlighting its suitability for complex plant matrices.

## 2 Results and Discussion

### 2.1 Characterization of 3D-CB/HEC–CH

Electrochemical characterization of the 3D-CB/HEC–CH electrode by CV in the [Fe­(CN)_6_]^3–/4–^ redox system revealed anodic and cathodic peaks with a peak-to-peak separation (ΔEp) larger than expected for a reversible process, indicating quasi-reversible/irreversible electron-transfer kinetics at the electrode surface. The anodic peak current showed a linear relationship with the square root of the scan rate between 10 and 100 mV s^–1^ (r > 0.998), confirming diffusion control, whereas the anodic peak potential (Ep) shifted linearly with log­(scan rate) (r = 0.980), consistent with the Nicholson–Shain treatment for irreversible systems.^30^ The slope of this relationship was used to determine the transfer coefficient (αn_a_).

Considering the irreversible redox behavior, a modified Randles–Ševčík equation incorporating the transfer coefficient was applied to calculate the electroactive surface area.^30^ A diffusion coefficient of 7.6 × 10^–6^ cm^2^ s^–1^ was used for the calculations, yielding an electroactive surface area of 0.37 cm^2^ for the modified electrode, consistent with values reported for carbon black 3D-printed electrodes.^31^ Considering a geometric area of 0.070 cm^2^, the corresponding roughness factor was estimated as 5.3, reflecting a 5.3-fold increase in active area relative to the geometric surface. These results indicate that surface modification led to a greater availability of electroactive sites at the electrode surface. The cyclic voltammograms, linear fitting, and calculation details are presented in Figure S1 (ESI).

The influence of surface modification on the charge-transfer properties of the electrodes was evaluated by EIS. Figure 2 presents the Nyquist plots for the (a) 3D-CB, (b) 3D-CB/HEC, (c) 3D-CB/CH, and (d) 3D-CB/HEC–CH electrodes. For the bare 3D-CB electrode, the semicircle diameter corresponded to a charge-transfer resistance (R_ct_) of 362.27 Ω. After modification with HEC, the R_ct_ increased to 495.98 Ω, indicating that this polymer layer introduces a barrier effect that hinders electron transfer. Modification with CH alone decreased the R_ct_ to 311.18 Ω, suggesting that chitosan provides a more favorable interface for charge transfer than HEC while maintaining electrode conductivity. This behavior can be attributed to the free amino groups of CH, which undergo protonation and establish electrostatic interactions with the negatively charged [Fe­(CN)_6_]^3–/4–^ redox probe, facilitating interfacial charge transfer.^26^

**Figure 2:**
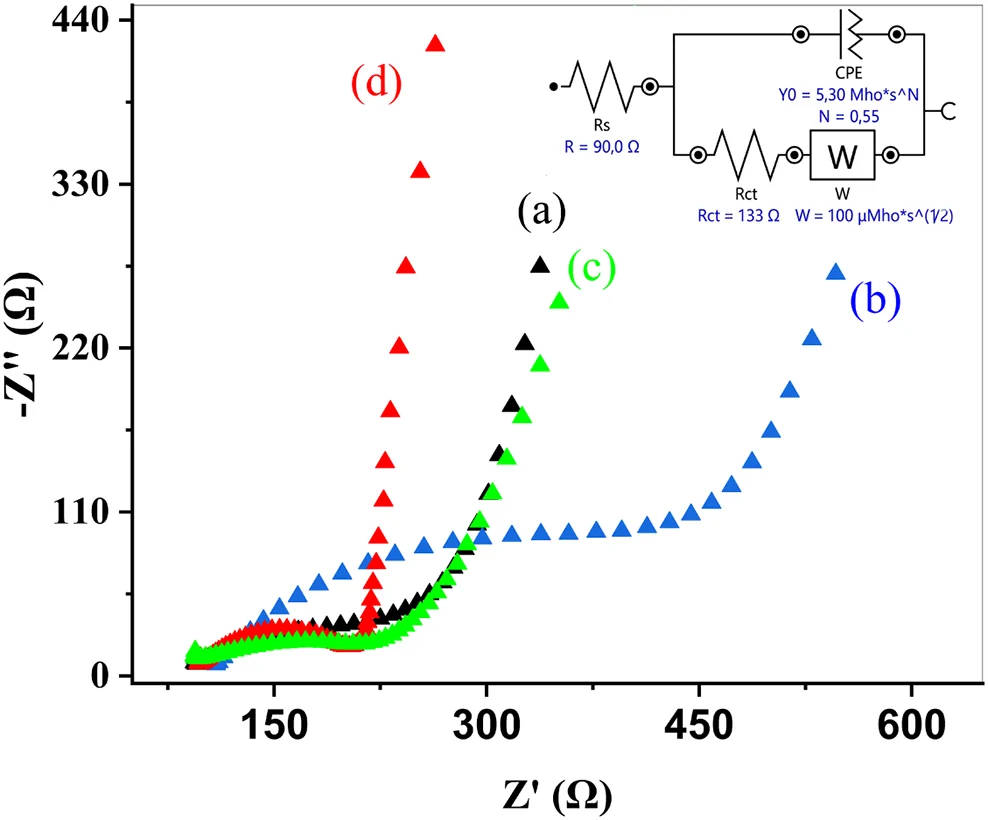
Nyquist plots recorded for (a) bare 3D-CB (black), (b) 3D-CB/HEC (blue), (c) 3D-CB/CH (green), and (d) 3D-CB/HEC-CH (red) electrodes in 0.020 M [Fe­(CN)_6_]^3–/4–^ containing 0.10 M KCl. EIS measurements covered frequencies from 100 kHz to 0.1 Hz, with a 10 mV sinusoidal perturbation applied at open-circuit potential.

The 3D-CB/HEC–CH electrode, in turn, exhibited a further decrease in R_ct_ to 132.82 Ω, indicating faster electron transfer than either polymer alone. This result suggests that HEC does not impair the electroactive function of CH within the composite film. This enhancement can be attributed to an improved organization of the polymeric matrix and electrostatic interactions between protonated CH amino groups and the negatively charged [Fe­(CN)_6_]^3–/4–^ species, facilitating charge transport. The higher CPE-Y_0_ value observed for 3D-CB/HEC–CH further indicates increased capacitance and charge accumulation. These findings are consistent with the electroactive area enhancement determined by CV. This behavior corroborates previous studies reporting enhanced charge-transfer properties for CH-modified electrodes.^32^

The actual contribution of HEC was further confirmed by comparing the signal stability of the 3D-CB/HEC–CH and 3D-CB/CH electrodes under prolonged exposure (Figure 3). Over five consecutive measurements with the same electrode continuously immersed in 0.50 M NaOH, the HEC–CH electrode exhibited stability (RSD of 4.77% for IAA and 4.34% for SA), whereas the CH-only electrode showed signal decline over the same period (RSD of 22.99% for IAA and 51.77% for SA), corresponding to a decrease of ∼61% in the SA signal between the first and last measurements, accompanied by a shift in the peak potential of both analytes. This is consistent with the hydrophilic, film-forming character of HEC, which promotes a more homogeneous matrix and may favor ion permeability, thereby stabilizing the CH film under prolonged exposure.^33^

**Figure 3:**
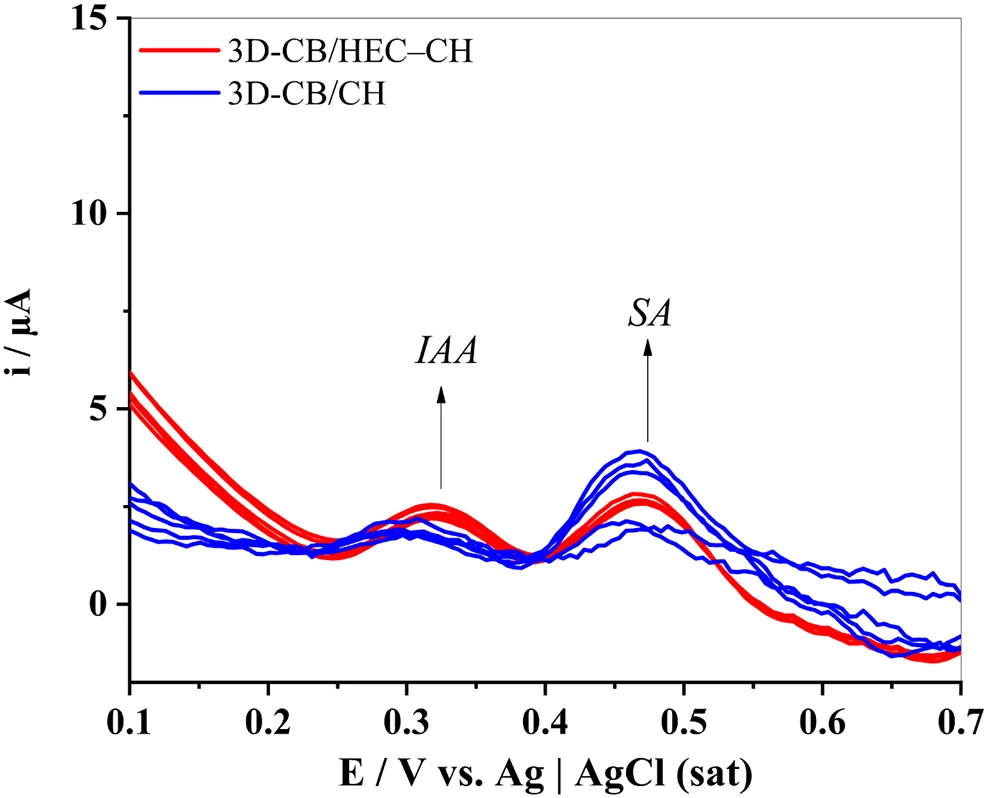
SWV responses obtained for five consecutive measurements at the (red) 3D-CB/HEC–CH and (blue) 3D-CB/CH electrodes, using the same electrode continuously immersed in sodium hydroxide solution (pH 13) containing IAA and SA, illustrating the signal stability of each surface under prolonged exposure. SWV parameters: a = 50 mV, f = 1 Hz, and (ΔE_s) = 0.5 mV.

Surface morphological features of the electrodes were examined by SEM, with micrographs presented in Figure 4a and c. SEM micrographs recorded at a 10 μm scale revealed that the 3D-CB electrode (Figure 4a) showed a granular morphology with interparticle voids, characteristic of the agglomerated structure of 3D-CB. The 3D-CB/HEC–CH-modified electrode (Figure 4c) showed a film-like structure covering the carbon particles. The blend was distributed over regions with surface relief, indicating successful deposition of the polymeric film. These morphological changes are consistent with the electrochemical results, including the increased electroactive surface area and lower R_ct_ values.

**Figure 4:**
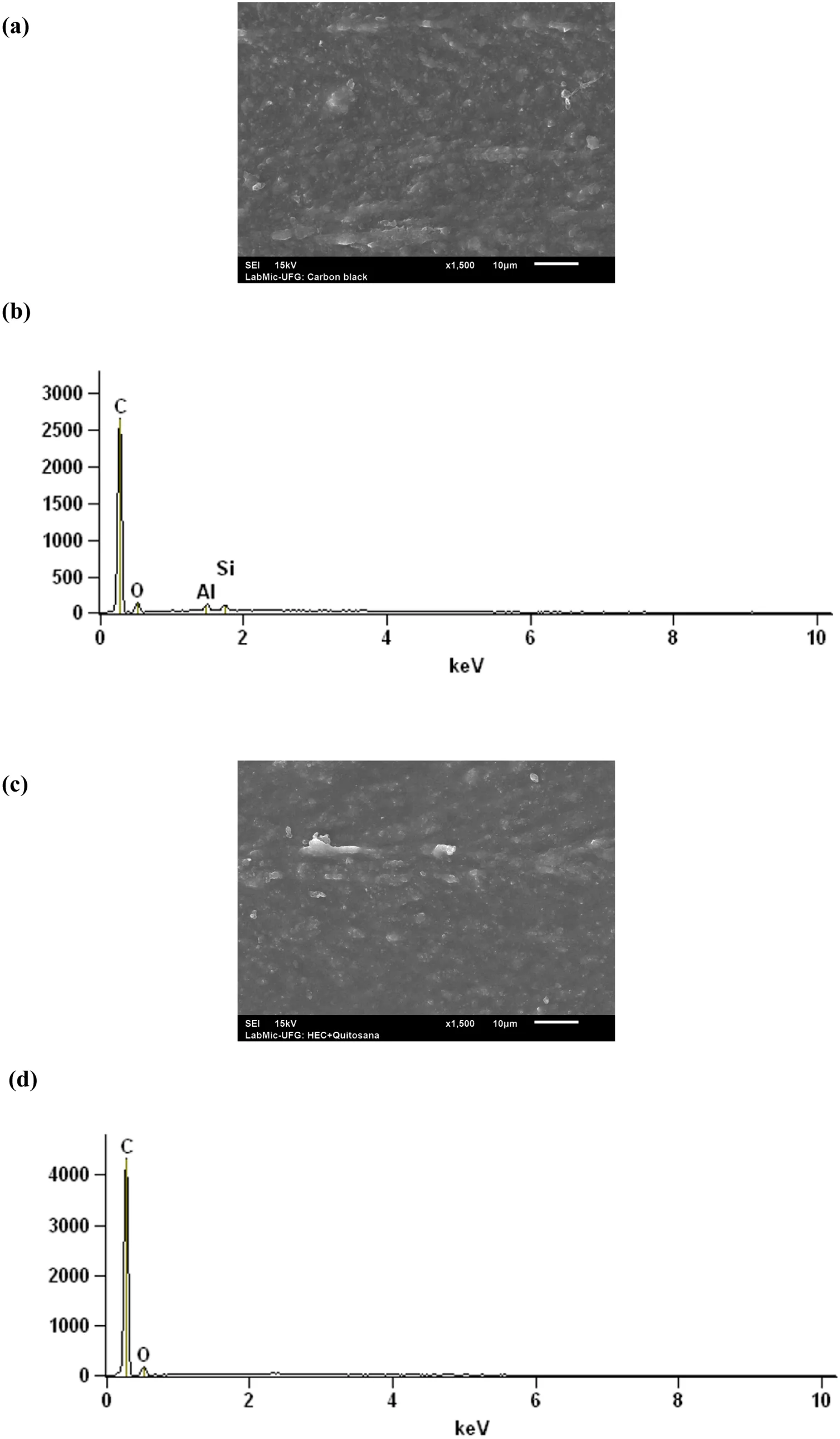
SEM micrograph (a) and the corresponding EDS spectrum (b) of the unmodified 3D-CB electrode, and SEM micrograph (c) and the corresponding EDS spectrum (d) of the 3D-CB/HEC–CH modified electrode. SEM images and EDS spectra were acquired at an accelerating voltage of 15.0 kV.

EDS analysis was performed to assess the surface elemental profile, with the corresponding spectra presented in Figure 4b and d. Although the SEM analysis suggested accumulation of the polymeric blend in regions with higher surface relief, the EDS spectra indicated a uniform coating distribution over the electrode surface. The spectrum of the unmodified 3D-CB electrode (b) showed major signals corresponding to carbon (C) and oxygen (O), along with trace amounts of aluminum (Al) and silicon (Si), which are most likely attributable to inorganic additives or residual mineral fillers present in the commercial CB/PLA filament used for electrode fabrication, as previously reported for conductive PLA-based filaments.^34^ These impurity signals were absent from the spectrum of the 3D-CB/HEC–CH-modified electrode (d).

To further assess compositional uniformity, EDS spectra were acquired from three different regions of each surface (Figures S2 and S3, ESI). The consistent C and O signals across all analyzed areas, together with the absence of additional elemental signals on the modified electrode, confirm the homogeneous distribution of the HEC–CH film over the electrode surface.

### 2.2 Optimization of Conditions for the 3D-CB/HEC–CH Electrode

The electrochemical responses of IAA and SA at the 3D-CB/HEC–CH electrode were evaluated as a function of pH using Britton–Robinson (BR) buffer over the pH range of 7–13. As shown in Figure S4 (ESI), the oxidation response of IAA was observed throughout the investigated pH range, with a progressive shift of the anodic peak toward less positive potentials as the pH increased. In contrast, SA exhibited well-defined oxidation peaks only at pH 7, 12, and 13, with no significant response observed at the remaining pH values, indicating that alkaline media were more favorable for the electrochemical response of both analytes. Overall, pH 13 was selected as the optimal condition since it provided the highest oxidation current for both analytes and a more effective separation between the anodic peaks of IAA and SA.

Based on the results obtained from the BR buffer study, the electrochemical behavior of IAA and SA was subsequently investigated in different supporting electrolytes, including 0.10 M PBS (pH 7), 0.10 M borate buffer (pH 9), and 0.50 M sodium hydroxide solution (pH 13). CVs were recorded at 50 mV s^–1^ between −1.50 and +1.00 V vs Ag|AgCl (sat.) at room temperature. Figure S5 (ESI) shows the cyclic voltammograms obtained for IAA and SA in the different supporting electrolytes evaluated.

Based on the cyclic voltammograms displayed in Figure S5 (ESI), recorded in 0.50 M sodium hydroxide solution (pH 13), a marked enhancement in the electrochemical response was observed for both IAA and SA when compared with the other investigated media. In this electrolyte, improved peak definition was achieved, indicating superior electrochemical sensitivity. Accordingly, the sodium hydroxide solution (pH 13) was selected as the supporting electrolyte for subsequent electrochemical analysis. Although forced degradation studies have reported that salicylic acid and related compounds can undergo alkaline hydrolysis under elevated temperatures and prolonged exposure,^35^ the electrochemical measurements in this study were performed on freshly prepared solutions over a short timescale at room temperature, conditions considerably milder than those employed in such degradation protocols.

The cyclic voltammograms displayed in Figure 5 show oxidation processes for both IAA and SA under the selected alkaline conditions. For IAA, oxidation begins at ∼+0.25 V, with a pronounced anodic response observed around +0.35 V vs Ag|AgCl (sat.). In contrast, SA exhibits an oxidation response at more positive potentials, with an anodic peak observed at approximately +0.65 V. The separation between the oxidation peak potentials enables the electrochemical discrimination of the two analytes during simultaneous analysis.

**Figure 5:**
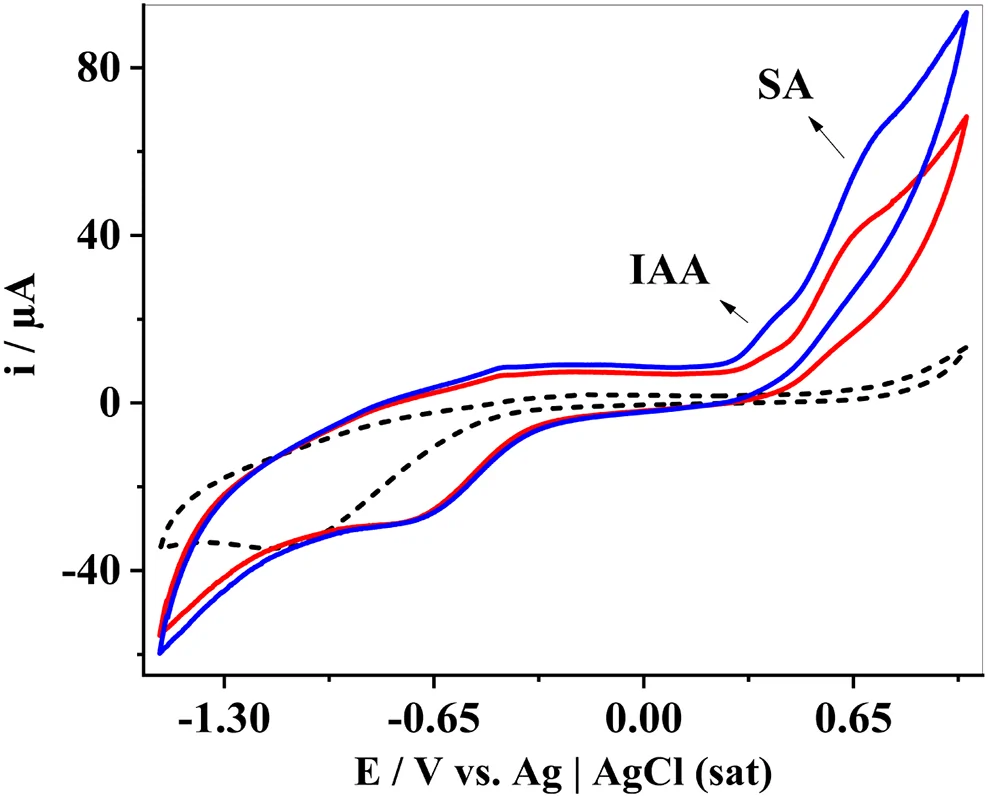
CV obtained at the 3D-CB/HEC–CH electrode for the simultaneous analysis of IAA and SA recorded in sodium hydroxide solution (pH 13), following the addition of the analytes at 50 μM (red) and 125 μM (blue). Scan rate: 50 mV s^–1^; step potential: 5 mV; potential window: −1.50 to +1.00 V vs Ag|AgCl (sat.).

The scan-rate data shown in Figure S6 (ESI) were analyzed to clarify the oxidation mechanism. For both analytes, the anodic peak current exhibited a linear dependence on the square root of the scan rate (r = 0.997 for IAA and r = 0.998 for SA). In addition, the plots of log Ip vs log ν (Figure S6a3 and b3 (ESI)) yielded slopes of 0.68 for IAA and 0.52 for SA. These results corroborate the finding that the oxidation of SA is predominantly diffusion-controlled, whereas the oxidation of IAA is mainly governed by diffusion with a minor contribution from adsorption.

The oxidation of IAA involves the transfer of two electrons and one proton, with formation of a 3-methyleneindolenine carboxylic acid cation as the principal oxidized species (eq 1).^36^ The dependence of the anodic peak potential on pH, evaluated using Britton–Robinson buffer (Figure S4b (ESI)), exhibited a slope of −56.3 mV pH^–1^, which supports the participation of protons in the oxidation process and is consistent with the proton-coupled electron-transfer mechanism previously reported for IAA. In turn, SA undergoes an electrochemical oxidation involving two electrons and two protons, leading to a quinone-like oxidized product (eq 2).^37^ As shown in Figure S4 c (ESI), SA exhibited a well-defined oxidation peak only under strongly alkaline conditions (pH ≥ 12); therefore, a systematic pH-dependent mechanistic evaluation could not be established for this analyte. Consequently, the proposed proton and electron stoichiometry for SA was assigned based on previous literature reports. These oxidation pathways account for the well-separated anodic peaks observed during simultaneous voltammetric measurements.

1 $$C_{10}H_{9}{\mathrm{NO}}_{2}\to C_{10}H_{8}{\mathrm{NO}}_{2}+{2e}^{-}+H^{+}$$

2 $$C_{7}H_{6}O_{3}\to C_{7}H_{4}O_{3}+{2e}^{-}+{2H}^{+}$$

### 2.3 Optimization of SWV Parameters and Analytical Signal Selection

SWV was employed for the simultaneous determination of IAA and SA in sodium hydroxide solution (pH 13), with the oxidation responses monitored between +0.10 and +0.80 V vs Ag|AgCl (sat.). The SWV parameters were optimized by evaluating modulation amplitude, frequency, and step potential using standard solutions containing 50 μM each of IAA and SA. The optimization results, including the evaluation of peak current, minimal full width at half-maximum (fwhm), and peak height (IP), are presented in Figures S7 and S8 (ESI). Based on the best compromise between peak intensity, peak definition, and simultaneous resolution of both analytes, the optimized SWV parameters were set to a modulation amplitude of 50 mV, a frequency of 1 Hz, and a step potential of 0.5 mV, which were used in the subsequent analytical studies.

### 2.4 Detection Performance of the 3D-CB/HEC–CH Electrode

The calibration of IAA and SA was assessed using triplicate measurements of standard solutions over the 12–87 μM concentration range. The resulting voltammograms and calibration plots are presented in Figure S9 (ESI), while the IP data are summarized in Table S1 (ESI). Least-squares regression was applied to the experimental data, producing individual calibration models for IAA and SA, each with r = 0.999.

The detection capability was evaluated according to standard criteria, yielding limits of detection (LODs) of 0.446 μM for IAA and 0.335 μM for SA, and limits of quantification (LOQs) of 1.359 μM and 1.018 μM, respectively. A linear relationship was observed for both analytes across the evaluated concentration interval, and the corresponding calibration equations for IAA (eq 3) and SA (eq 4) are presented below:

3 $$i\left(\mathrm{\mu A}\right)=\left(0.281\pm 0.024\right)+\left(0.032\pm 0.001\right)\times C_{\mathrm{IAA}}\left(\mathrm{\mu M}\right)\mathrm{; r}=0.999$$

4 $$i\left(\mathrm{\mu A}\right)=\left(1.265\pm 0.077\right)+\left(0.105\pm 0.001\right)\times C_{\mathrm{SA}}\left(\mathrm{\mu M}\right)\mathrm{; r}=0.999$$

The data met the assumptions of normality and homoscedasticity. The lack-of-fit test for the calibration models showed no deviation from linearity across the evaluated concentration range. These statistical findings support the selectivity of the 3D-CB/HEC–CH electrode and demonstrate the suitability of the proposed method for the simultaneous determination of IAA and SA in plant-based samples.

The stability of the SWV response obtained with the 3D-CB/HEC–CH electrode was assessed at two concentration levels within the calibration range, namely 12 and 62 μM for both IAA and SA. At each concentration, eight consecutive measurements (n = 8) were performed in a single day to assess intraday precision, whereas interday precision was evaluated using measurements collected after a 48 h interval (raw data are provided in Table S2, ESI). The intra- and interday mean responses were compared at each concentration using one-way ANOVA (α = 0.05). The results are summarized in Table 1.

**Table 1:** Intraday and Interday Precision and Stability of the 3D-CB/HEC–CH SWV Method at Two Concentration Levels within the Calibration Range (12 and 62 μM IAA and SA)

IAA Concentration (μM)	Series	Mean (μA)	SD (μA)	RSD (%)
12	Intraday	0.96	0.01	0.99
12	Interday	0.95	0.03	3.30
62	Intraday	5.74	0.10	1.79
62	Interday	5.82	0.11	2.02
SA Concentration (μM)	**Series**	**Mean (μA)**	**SD (μA)**	**RSD (%)**
12	Intraday	0.42	0.02	4.64
12	Interday	0.40	0.02	4.27
62	Intraday	5.58	0.19	3.65
62	Interday	5.45	0.08	1.62
ANOVA summary (intra- vs interday)				
IAA Concentration (μM)	**F-value**	**p-value**	**Decision (α = 0.05)**	
12	0.62	0.44	Not significant	
62	2.29	0.15	Not significant	
SA Concentration (μM)	**F-value**	**p-value**	**Decision (α = 0.05)**	
12	0.41	0.53	Not significant	
62	0.01	0.89	Not significant	

For IAA and SA, the 3D-CB/HEC–CH electrode exhibited satisfactory intraday and interday precision, with RSD values below 4.70% (Table 1). The obtained values were consistent with the criteria established by the Association of Official Analytical Chemists (AOAC).^38^ The low variability observed across the measurements indicates good response stability and suggests the absence of a memory effect at the electrode surface, including during alternating injections of low- and high-concentration solutions.

Figure S10 (ESI) shows the voltammetric responses recorded in triplicate for a standard solution containing 25 μM IAA and SA, using three independently prepared electrodes, as an additional assessment of intra- and interday precision. The RSD values obtained were 4.55% for IAA and 4.49% for SA. One-way ANOVA revealed a statistically significant difference among electrodes for IAA (F = 9.51, p = 0.0138), whereas no significant difference was observed for SA (F = 1.37, p = 0.323). Nevertheless, each electrode individually exhibited good precision, with RSD values within acceptable limits across the triplicate measurements, indicating that the observed variability arose from small differences between electrodes rather than from inconsistent performance of any single electrode.

A study was conducted to evaluate the mutual influence of IAA and SA within the working range of the electrode. In this experiment, the maximum concentration of each compound (87 μM) was introduced into the system, followed by progressive additions to achieve a 1:1 ratio of the analytes. The goal was to determine whether the presence of one analyte would significantly affect the anodic signal of the other (Figure S11 (ESI)).

The mutual influence of IAA and SA on each other’s anodic signals was evaluated within the working range up to a 1:1 ratio of the analytes (Figure S11, ESI). Signal recovery remained within ±10% throughout the tested range; above 87 μM, however, larger deviations were observed, supporting the selection of this concentration as the upper limit of the working range and confirming accurate simultaneous determination of IAA and SA without significant mutual interference. These results demonstrate that the 3D-CB/HEC–CH electrode can reliably measure IAA and SA when both are present, within the established working range, including up to the upper limit of 87 μM, confirming that simultaneous detection of both compounds is feasible in complex plant samples (data reported in Table S3, ESI).

In addition, tests were conducted with other interfering compounds to evaluate the reliability of the 3D-CB/HEC–CH electrode in plant samples. The influence of potential interferents on the SWV responses of IAA and SA (87 μM) was evaluated under optimized conditions. Each species was analyzed in a 1:1 ratio with IAA and SA: phenolic compounds (gallic acid and catechol), organic acids (benzoic acid and citric acid), antioxidant compounds (ascorbic acid and rutin), sugars (sucrose), an amino acid (l-phenylalanine (Phe)), and phytohormones (kinetin, indole-3-butyric acid (IBA), and abscisic acid (ABA)). In all cases, the anodic peak current (IAA, ∼+0.28 V; SA, ∼+0.45 V) varied by ≤5% relative to the signals of the pure compounds (Figure 6). The raw triplicate data used for these analyses are presented in Table S4 (ESI).

**Figure 6:**
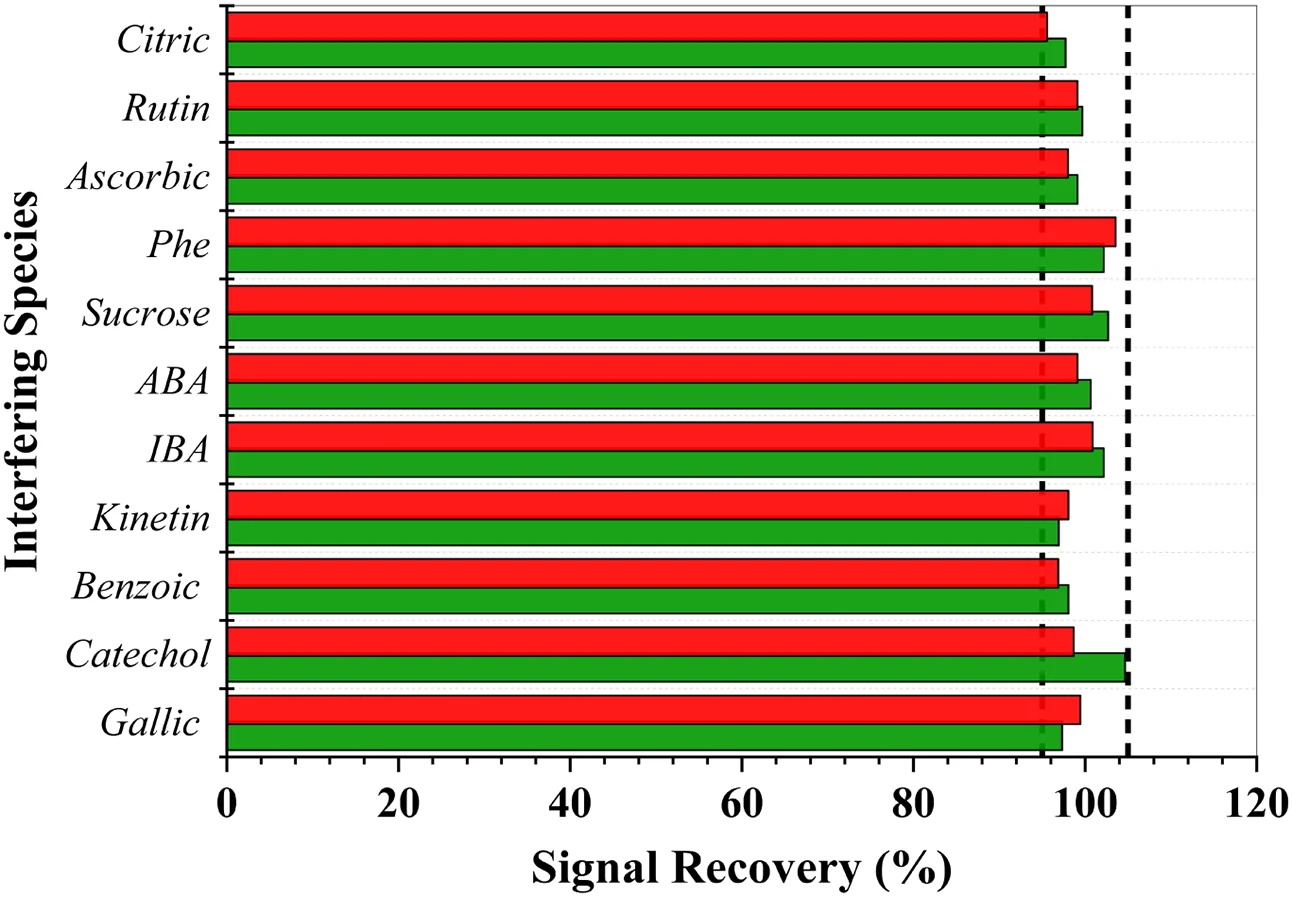
Influence of potential interferents on the peak current responses of IAA and SA (87 μM) in sodium hydroxide solution (pH 13) as the supporting electrolyte. The ratio of IAA and SA to the interfering agent (1:1) was used. The dashed black band marks ±5% (vertical black lines). All values lay within this range. SWV parameters: a = 50 mV, f = 1 Hz, and (ΔE_s) = 0.5 mV.

The assumptions of normality and homoscedasticity were satisfied for all groups, with no significant deviations observed based on the corresponding p-values. One-way ANOVA revealed no statistically significant differences among the group means at a significance level of α = 0.05.

The robustness of the method was examined by independently varying temperature, operator, and supporting electrolyte concentration while maintaining the remaining experimental conditions unchanged. None of these factors produced a statistically significant effect on the analytical response, based on triplicate measurements (Table S5, ESI).

Overall, the 3D-CB/HEC–CH-based SWV method exhibited satisfactory linearity, selectivity, precision, and robustness, supporting its applicability to the analysis of real plant samples. The main analytical performance characteristics obtained during validation are compiled in Table 2.

**Table 2:** Validation Results for the Simultaneous SWV Determination of IAA and SA with the 3D-CB/HEC–CH Electrode

Analytical Parameters	Results IAA	Results SA
Linear range (μM)	12–87	12–87
r	0.999	0.999
LOD (μM)	0.446	0.335
Lack-of-fit	0.973 (*p* > 0.05)	0.973 (*p* > 0.05)
Intraday RSD	0.99% (12 μM) – 1.79% (62 μM)	4.64% (12 μM) – 3.65% (62 μM)
Interday RSD	3.30% (12 μM) – 2.02% (62 μM)	4.27% (12 μM) – 1.62% (62 μM)
Robustness (Δsignal)	3.97%	0.70%

### 2.5 Real Sample Analysis

The applicability of the proposed SWV method, using the modified electrode, was evaluated for the quantification of IAA and SA in real plant matrices. Root and leaf samples of soybean and maize, as well as root, leaf, and green fruit samples of strawberry, were analyzed under the optimized experimental conditions.

The plant samples were subjected to the established extraction procedure, and the extracts were analyzed before and after fortification using the standard-addition method. The additions were performed within the linear concentration range established for each analyte, and the net peak currents are presented in Table S6 (ESI). Calibration plots were generated by applying least-squares linear regression to the complete set of addition levels. Each determination was carried out in triplicate. The corresponding calibration equations and correlation coefficients are summarized in Table 3, and the recorded SWV voltammograms (Figure 7) show well-defined oxidation peaks for both IAA and SA in the plant extracts.

**Table 3:** Calibration Parameters Obtained by the Standard-Addition Method for IAA and SA in Plant Matrices

Sample	Analyte	Calibration equation	r
Soybean	IAA	i (μA) = (0.208 ± 0.062) + (0.023 ± 0.001) × C (μM)	0.998
Soybean	SA	i (μA) = (0.057 ± 0.041) + (0.052 ± 0.001) × C (μM)	0.999
Maize	IAA	i (μA) = (0.235 ± 0.013) + (0.020 ± 0.001) × C (μM)	0.999
Maize	SA	i (μA) = (0.295 ± 0.011) + (0.048 ± 0.001) × C (μM)	0.999
Strawberry	IAA	i (μA) = (1.783 ± 0.048) + (0.042 ± 0.001) × C (μM)	0.999
Strawberry	SA	i (μA) = (2.456 ± 0.133) + (0.141 ± 0.002) × C (μM)	0.999

**Figure 7:**
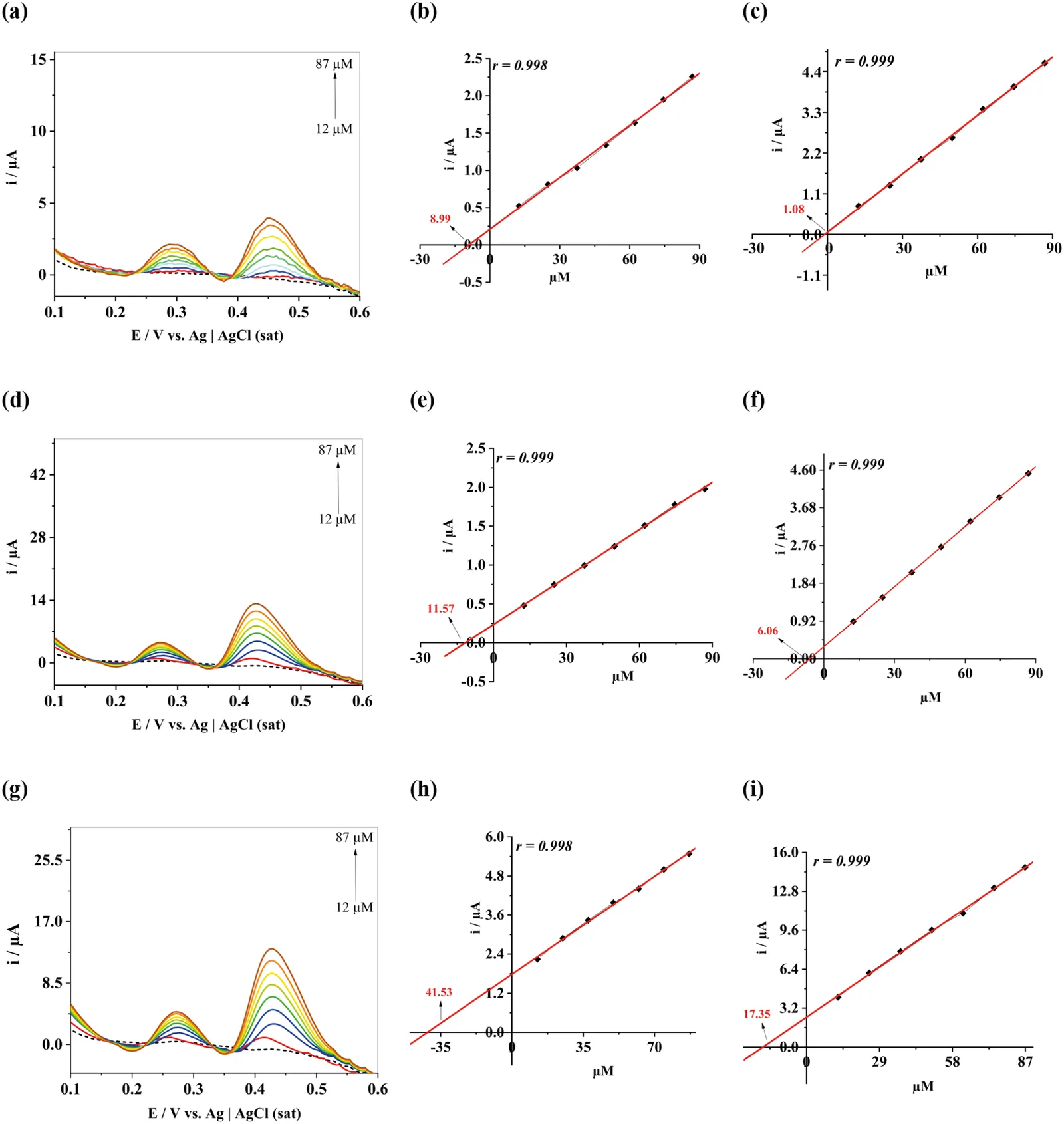
Baseline-corrected SWV responses (*n* = 3) obtained in plant extracts using the standard-addition method. The supporting electrolyte (black lines) and plant extracts (red) are indicated in the voltammetric profiles. (a–c) Soybean extract: (a) voltammograms; (b) IAA calibration curve; (c) SA calibration curve; (d–f) Maize extract: (d) voltammograms; (e) IAA calibration curve; (f) SA calibration curve; (g–i) Strawberry extract: (g) voltammograms; (h) IAA calibration curve; (i) SA calibration curve. SWV parameters: see Figure 6.

The proposed method enabled the quantification of endogenous phytohormones in plant tissue extracts, yielding concentrations of 8.99 μM of IAA and 1.08 μM of SA in soybean, 11.57 μM of IAA and 6.06 μM of SA in maize, and 41.53 μM of IAA and 17.35 μM of SA in strawberry extracts, which exhibited the highest phytohormone levels among the analyzed samples. The endogenous concentrations of IAA and SA in plant extracts were determined by the standard-addition method to minimize matrix effects.

Spike-recovery assays were used to assess the accuracy of the proposed method in real plant extracts. The samples were fortified with known amounts of IAA and SA, followed by electrochemical measurements under the optimized conditions. The concentrations of the fortified samples were calculated using the corresponding standard-addition calibration equations for each extract. Recovery (%) was calculated as the ratio of the increase in the measured concentration after fortification to the added concentration of each analyte.

The accuracy of IAA and SA determinations was assessed using soybean, maize, and strawberry extracts fortified with 25 and 75 μM for each analyte (n = 3). Electrochemical measurements were conducted under the optimized SWV/3D-CB/HEC–CH conditions, with the results presented in Table 4.

**Table 4:** Spike–Recovery Results for IAA and SA in Soybean, Maize, and Strawberry Extracts at 25 and 75 μM, Obtained under the Optimized SWV/3D-CB/HEC–CH Conditions (n = 3)

Sample	Analyte	Added (μM)	Spike recovered (μM mean ± SD)	Recovery (%)	RSD (%)
Soybean	IAA	25.00	26.99 ± 0.74	99.31	2.74
Soybean	IAA	75.00	74.87 ± 1.95	97.51	3.18
Soybean	SA	25.00	25.74 ± 0.76	102.98	2.97
Soybean	SA	75.00	74.69 ± 2.60	100.19	3.49
Maize	IAA	25.00	26.30 ± 1.01	96.82	3.85
Maize	IAA	75.00	75.69 ± 3.21	98.71	5.27
Maize	SA	25.00	26.39 ± 1.06	99.95	4.01
Maize	SA	75.00	74.57 ± 2.21	98.13	2.72
Strawberry	IAA	25.00	37.00 ± 1.19	97.96	3.21
Strawberry	IAA	75.00	88.09 ± 1.59	101.37	3.33
Strawberry	SA	25.00	25.66 ± 0.83	98.83	3.24
Strawberry	SA	75.00	75.08 ± 0.62	99.43	1.92
			**Across-level mean**	**99.27 ± 1.71**	**1.72**

For IAA, recoveries ranged from 96.82% to 101.37%, with RSD values between 2.74% and 5.27%. For SA, recoveries ranged from 98.13% to 102.98%, with RSD values between 1.92% and 4.01% (Table 4). Considering all fortification levels and plant matrices, the overall mean recovery was 99.27 ± 1.71%, with an RSD of 1.72%. These results demonstrate adequate precision and accuracy and indicate that the sample matrices did not significantly interfere with the determination of either analyte. The raw triplicate data used for the recovery calculations are provided in the Supporting Information (Table S7, ESI).

A two-tailed Student’s *t*-test was applied to evaluate the trueness of the results by comparing theoretical and experimentally determined concentrations. At the 95% confidence level, all calculated values were lower than the critical value, indicating no statistically significant difference between added and found concentrations. The recovery results indicate that the proposed approach provides reliable determination of IAA and SA in soybean, maize, and strawberry samples.

Table 5 summarizes a comparison between the proposed approach and analytical methods previously described for the determination of IAA and SA. Although many electrochemical sensors described in the literature exhibit high sensitivity, they rely on elaborate architectures and nanomaterials, which may hinder routine application. As shown in Table 5, these platforms commonly employ carbon nanotubes,^1,16,39^ MXenes, molecularly imprinted polymers,^40^ gold nanoparticles,^41^ and other nanostructured materials^7^ that require multistep fabrication and surface modification procedures. In contrast, the proposed 3D-CB/HEC–CH sensor employs a simple HEC–CH biopolymer bilayer deposited onto a 3D-printed carbon electrode through a straightforward modification process, eliminating the need for nanostructured materials while enabling reliable simultaneous determination of IAA and SA.

**Table 5:** Comparison of Analytical Approaches Reported for the Simultaneous Determination of IAA and SA, Including the Proposed Method^a^

Technique	LOD (μM)	r	Linear Range (μM)	Sample Matrix	Electrode Type	Ref.
LC-ESI-MS/MS	0.055/0.001	0.999/0.999	0.29–1.71	tomato	-	^8^
HF-LLLME-HPLC-UV	0.007/0.026	0.999/0.999	0.15–14.48	natural coconut juice	-	^9^
DPV	1.990/3.300	0.993/0.974	25–1000	soybean seedlings	CB–MWNT–Nafion/Fc/CB–MWNT/GC	^1^
DPV	0.010/0.010	0.988/0.974	0.01–100	*Arabidopsis thaliana* leaves	Electrodes modified with carbon cement	^7^
DPV	0.100/0.100	0.996/0.995	0.10–20.0	tomato leaves	MWCNTS/CCC/SS	^16^
DPV	0.921/1.677	0.995/0.994	0.05–25.0	strawberry pulp	MIP/MXene/LIG/PDMS	^40^
LSV	0.007/0.100	0.997/0.997	5.0–100	rape leaves and broad bean	PPRONPs–CDs–MWCNTs	^39^
CA	0.210/0.220	0.978/0.996	0.80–188.4	artificial samples	AuNPs-GH/GCEs	^41^
SWV	0.446/0.335	0.999/0.999	**12–87**	**multiple plant tissues**	**3D-CB/HEC–CH**	**This work**

a LC-ESI-MS/MS (Liquid Chromatography–Electrospray Ionization–Tandem Mass Spectrometry); HF-LLLME-HPLC-UV (Liquid–Liquid Microextraction Coupled with High-Performance Liquid Chromatography with UV Detection); DPV (Differential Pulse Voltammetry); LSV (Linear Sweep Voltammetry); CA (Chronoamperometry); SWV (Square-Wave Voltammetry).

Although chromatographic techniques^8–10^ generally offer wider linear ranges and lower detection limits compared with electrochemical methods, as demonstrated by the LODs and linear ranges presented in Table 5, they require expensive instrumentation, specialized infrastructure, and laborious sample preparation, limiting large-scale monitoring. Electrochemical sensors, in turn, offer the advantages of faster analysis, simpler instrumentation, and lower operational costs, making them attractive for large-scale and field-based applications.

The proposed method employs biodegradable polymeric materials and a simple 3D-CB electrode architecture, using low-impact solvents in accordance with green analytical principles.^42^ The simultaneous determination of IAA and SA was achieved using SWV, a rapid and sensitive technique that remains scarcely explored for the simultaneous detection of these phytohormones. The fast signal acquisition and reduced analysis time, inherent to SWV, further enhance the practical applicability of the proposed method. Together with the straightforward fabrication of both the 3D-printed electrode and the HEC–CH surface modification, as well as reduced operational cost, the proposed platform is easily scalable, offering a practical, sustainable, reproducible, and time-efficient alternative for the routine monitoring of IAA and SA in plant matrices.

Accordingly, the proposed sensor is best regarded as a complementary tool for rapid, low-cost screening applications, whereas chromatographic and mass spectrometry-based techniques remain more suitable for confirmatory analysis or scenarios requiring detection at submicromolar concentrations.

## 3 Conclusion

In this work, a 3D-CB/HEC–CH electrode was successfully developed for the simultaneous voltammetric detection of IAA and SA, combining low-cost 3D-printed electrodes with a biodegradable HEC–CH bilayer, without the need for nanomaterials or multistep surface modifications. The method exhibited excellent linearity (r = 0.999), low detection limits (0.446 μM for IAA and 0.335 μM for SA), and good precision (RSD < 4.70%) and was successfully validated in soybean, maize, and strawberry matrices, with recoveries between 96.82 and 101.37% for IAA and 98.13 and 102.98% for SA, confirming its reliability across tissues with different compositions.

Compared with chromatographic techniques, the proposed method offers faster analysis, simpler instrumentation, and lower operational costs, making it particularly well-suited for rapid screening applications, whereas trace-level confirmatory analysis remains more appropriate for chromatographic and mass spectrometry-based approaches. Future work should focus on testing the electrode under field conditions and with a broader range of plant species, as well as exploring its integration into portable or automated platforms. Overall, the 3D-CB/HEC–CH electrode constitutes a sustainable, low-cost, and field-deployable alternative for decentralized phytohormone monitoring, with promising potential for application in precision agriculture.

## 4 Methods

### 4.1 Reagents and Samples

The chemicals used in this study were purchased from the following suppliers: HEC from Quimisul (São Paulo, SP, Brazil); boric acid, sodium hydroxide, and ascorbic acid from Alphatec (São Paulo, SP, Brazil); potassium ferricyanide, potassium chloride, CH, IAA, abscisic acid (ABA), indole-3-butyric acid (IBA), and kinetin from Sigma-Aldrich (St. Louis, MO, USA); potassium ferrocyanide, SA, and benzoic acid from Exodo Científica (Hortolándia, SP, Brazil); ethyl alcohol from ACS Científica (São Paulo, SP, Brazil); sodium phosphate monobasic, sodium phosphate dibasic, phosphoric acid, citric acid, and catechol from Neon (Suzano, SP, Brazil); acetic acid and gallic acid from Dinâmica (Indaiatuba, SP, Brazil); and l-phenylalanine from Synth (Diadema, SP, Brazil). All solutions were prepared using distilled water (resistivity ≥ 18 MΩ·cm), except for the IAA, SA, ABA, IBA, and kinetin standard solutions, which were prepared in ethanol. All chemicals were of analytical grade and were used as received, without further treatment.

The influence of pH on the electrochemical response of IAA and SA was first evaluated using a 0.1 M Britton–Robinson (BR) buffer solution, prepared from acetic, boric, and phosphoric acids mixed in equimolar proportions, which covers a pH range of 7 to 13. Reported structural alterations in CH under acidic conditions precluded the evaluation of lower pH values.^28^ BR buffer, PBS, and borate buffer solutions were selected for subsequent evaluation as supporting electrolytes, with their pH adjusted using 0.5 M sodium hydroxide. These pH values were selected based on the acid–base properties of IAA and SA, since their protonation state directly affects their oxidation mechanism and electron-transfer kinetics. Under alkaline conditions, progressive deprotonation of the carboxylic groups in both analytes, as well as the phenolic group in SA, alters their voltammetric response, which justifies exploring alkaline media to characterize their electrochemical behavior.^29^

Stock standard solutions of IAA and SA were prepared at a concentration of 0.025 M in ethanol and used to construct separate analytical calibration curves for each analyte. For the simultaneous determination of both analytes and for the standard addition experiments, mixed standard solutions were prepared at a concentration of 0.0125 M for each compound in the same medium.

To evaluate the applicability of the proposed sensor, plant samples were analyzed using the standard addition method to minimize potential matrix effects. Soybean and maize roots and leaves, as well as strawberry roots, leaves, and fruits, were collected from a rural property located in Rio Verde, Goiás, Brazil. Approximately 300 g of each plant tissue was macerated overnight in 30 mL of ethanol. The mixture was subjected to ultrasonic extraction (40 kHz) for 30 min at room temperature. Following extraction, the samples underwent centrifugation at 5000 × *g* for 30 min. The resulting supernatant was then collected for electrochemical analysis. For the electrochemical assays, 0.1 mL of the obtained extract was added to the electrochemical cell containing the supporting electrolyte.

### 4.2 Instrumentation and Equipment

All electrochemical experiments were conducted at ambient temperature under dissolved oxygen conditions with a PGSTAT302N potentiostat/galvanostat (Eco Chemie, Metrohm, Utrecht, Netherlands) operated via NOVA 2.1.7. Both cyclic voltammetry (CV) and SWV were performed using a three-electrode configuration comprising a 3D-CB/HEC–CH working electrode, a platinum wire auxiliary electrode (Metrohm, Utrecht, Netherlands), and an Ag/AgCl reference electrode (sat. KCl; Metrohm, Utrecht, Netherlands).

CB/PLA conductive filament (Protopasta, Vancouver, WA, USA) was used to produce the 3D-CB electrodes with a Creality Ender 2 Pro printer (Shenzhen, Guangdong, China). Each electrode measured 50 mm in length, with outer and inner diameters of 4.60 and 3.31 mm, respectively. The electrode geometry was designed in Autodesk Fusion and converted into printable files using Creality Slicer. The design file is included in the Supporting Information (Figure S12, ESI) to facilitate reproduction of the fabrication procedure. Surface preparation consisted of sequential polishing with 600- and 1200-grit sandpaper using ultrapure water.

### 4.3 Preparation and Characterization of 3D-CB/HEC–CH

Prior to each measurement, the 3D-printed carbon electrode was mechanically polished and electrochemically activated, following a protocol adapted from the literature.^21^ For film preparation, 1% (w/v) stock solutions of HEC and CH were used. The CH solution was prepared by dissolving CH in 0.10 M acetic acid, whereas water was used as the solvent for HEC. The bilayer was deposited by drop-casting: 10 μL of HEC solution was first applied onto the electrode surface and dried at 40 °C for 24 h, after which 10 μL of CH solution was applied and dried under the same conditions.^27^ This renewal protocol, comprising polishing, activation, and fresh HEC–CH deposition, was performed daily prior to each set of measurements, ensuring reproducible electrode surfaces while minimizing degradation of the biopolymer film upon prolonged exposure to the alkaline supporting electrolyte.

Electrode characterization was performed by CV using a 0.020 M [Fe­(CN)_6_]^3–/4–^ solution prepared in 0.10 M KCl. Voltammograms were recorded between −0.2 and +0.7 V at scan rates ranging from 10 to 100 mV s^–1^, with a potential step of 1 mV. The resulting data were used to estimate the electroactive surface area using a modified Randles–Ševčík equation incorporating the transfer coefficient, considering the observed irreversible electron-transfer behavior.

Electrochemical impedance spectroscopy (EIS) was conducted in the same redox probe medium at the formal potential, using an AC amplitude of 10 mV and frequencies from 100 kHz to 0.1 Hz.

Scanning electron microscopy (SEM) was used to examine the electrode morphology using a JSM-6610 microscope (JEOL, Tokyo, Japan). The surface elemental composition was determined by energy-dispersive X-ray spectroscopy (EDS) using a detector coupled to the microscope and a Thermo Scientific NSS Spectral Imaging system.

SWV was used for the simultaneous detection of IAA and SA under optimized experimental conditions.

## Supplementary Material



## Data Availability

The data supporting this article have been included as part of the Supporting Information.

## Notes

Figures S1–S12, containing cyclic voltammograms for electroactive area estimation, EDS spectra, pH and supporting electrolyte optimization studies, scan-rate effects, SWV parameter optimization, calibration voltammograms, reproducibility studies, mutual interference assays, and the design figure and file for electrode fabrication; and Tables S1–S7, providing raw data for calibration, precision, interference, robustness, and recovery analyses (PDF)
