# Supplementary material for: Hydroxyethylcellulose- and Chitosan-Modified 3D-Printed Carbon Electrodes for Indole-3-Acetic Acid and Salicylic Acid Determination
Source: ACS Omega. 2026 Sep 21;11(39):60147–59. doi: 10.1021/acsomega.6c07701 (PMC13647872; doi:10.1021/acsomega.6c07701)
Supplement: Supplementary file 1 [file ao6c07701_si_001.pdf]

Hydroxyethylcellulose and chitosan modified 3D-printed carbon electrodes for indole-3-acetic and salicylic acid determination

Pablo H. S. Martins *[a], Hugo L. S. L. Leão [a] and Polyana F. Pereira [a]

[a.] *Instituto Federal Goiano – Campus Rio Verde - Rod. Sul Goiana Km 01. Zona Rural. Rio Verde - GO – Brazil, 75901-970.*

(a) **(b)**

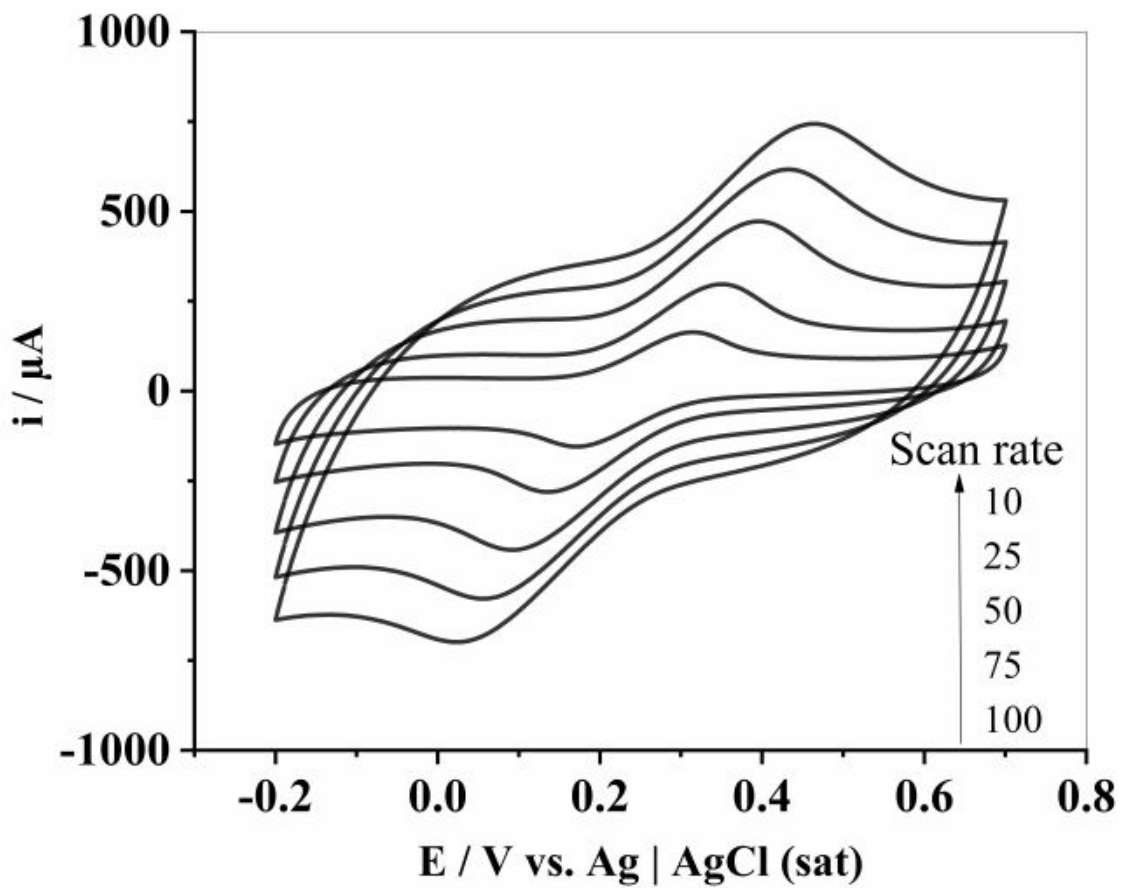

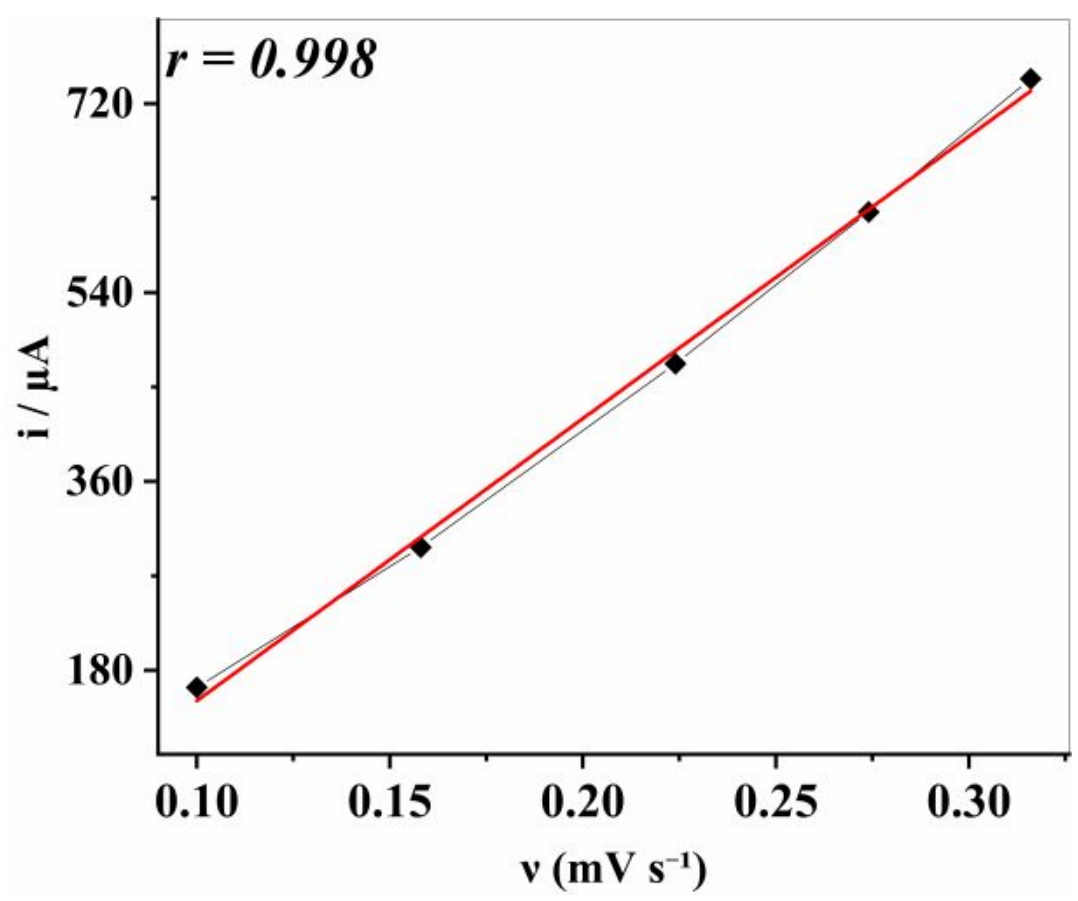

Figure S1. (a) Cyclic voltammograms of the 3D-CB/HEC-CH electrode recorded in 0.02 M $[Fe(CN)_6]^{3-/4-}$+ 0.10 M KCl at scan rates ranging from 10 to 100 mV s⁻¹. (b) Linear dependence of the anodic peak current on the square root of the scan rate used for electroactive area estimation. The measurements were performed in the potential window from −0.2 to +0.7 V using a potential step of 1 mV.

(a)

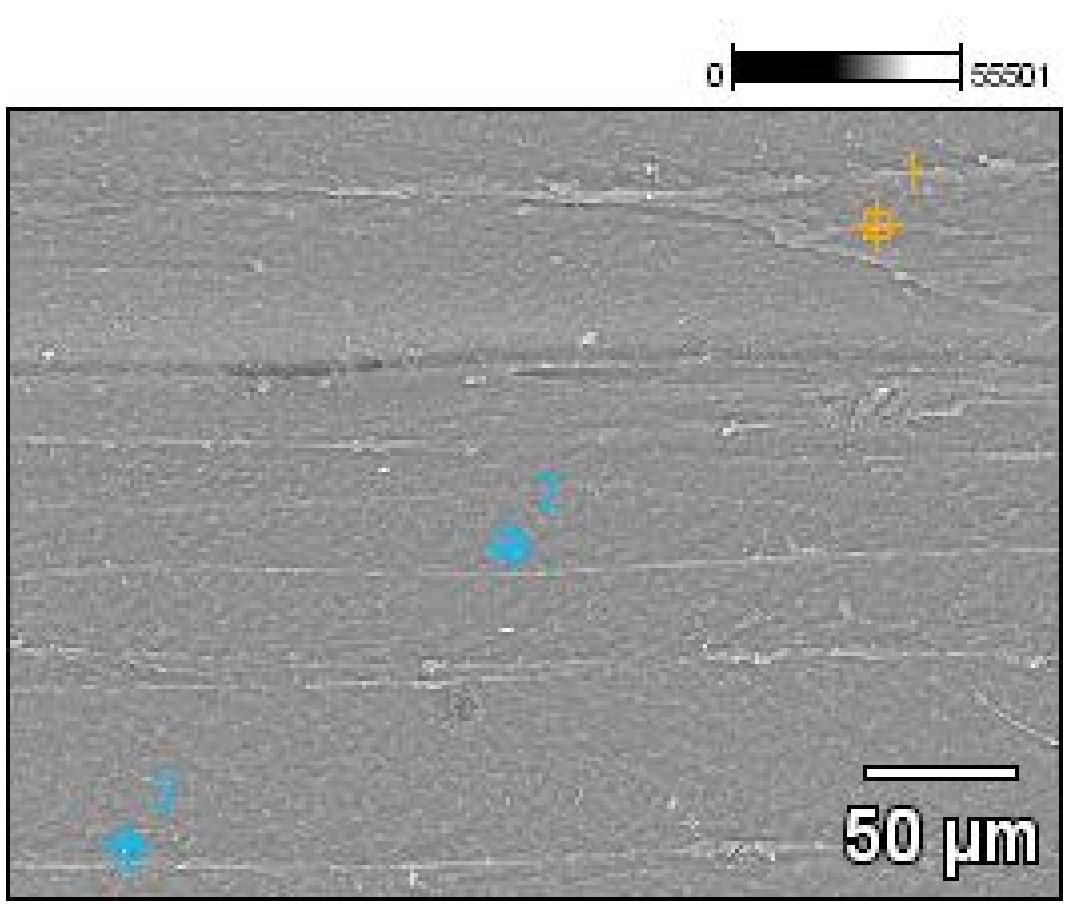

(a1)

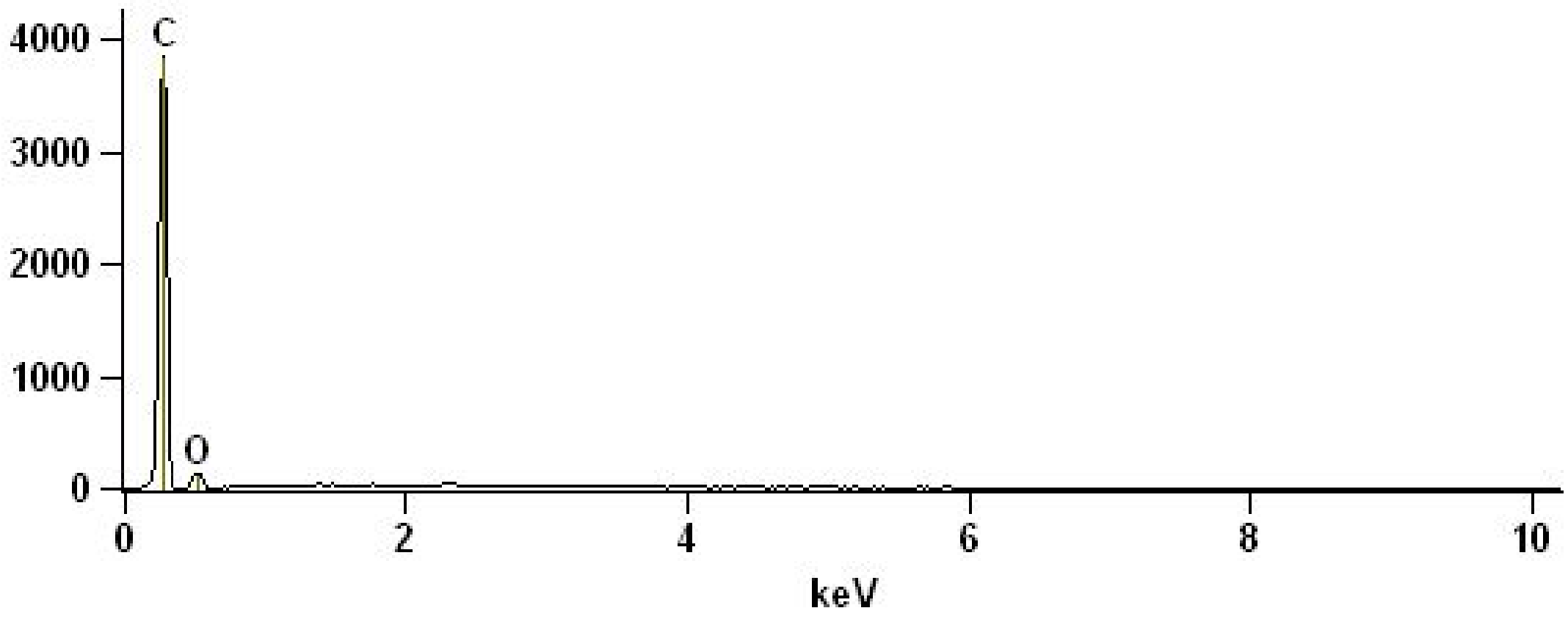

(a2)

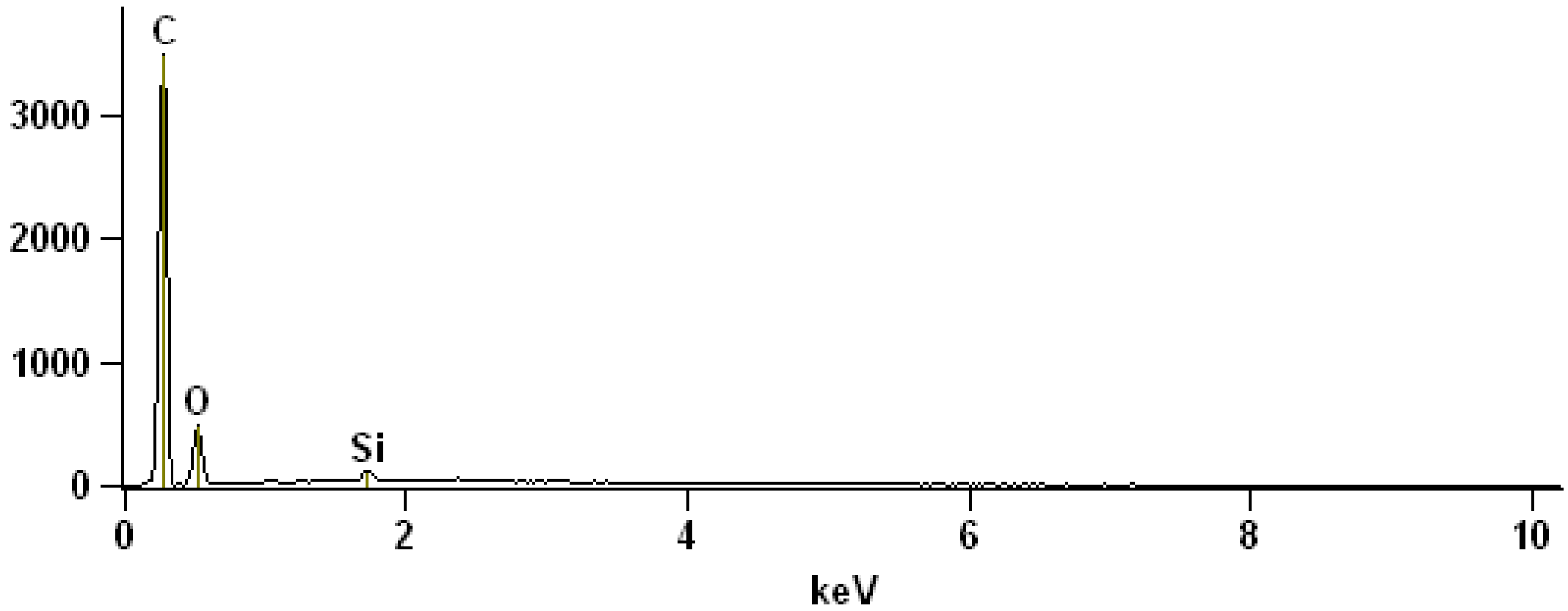

(a3)

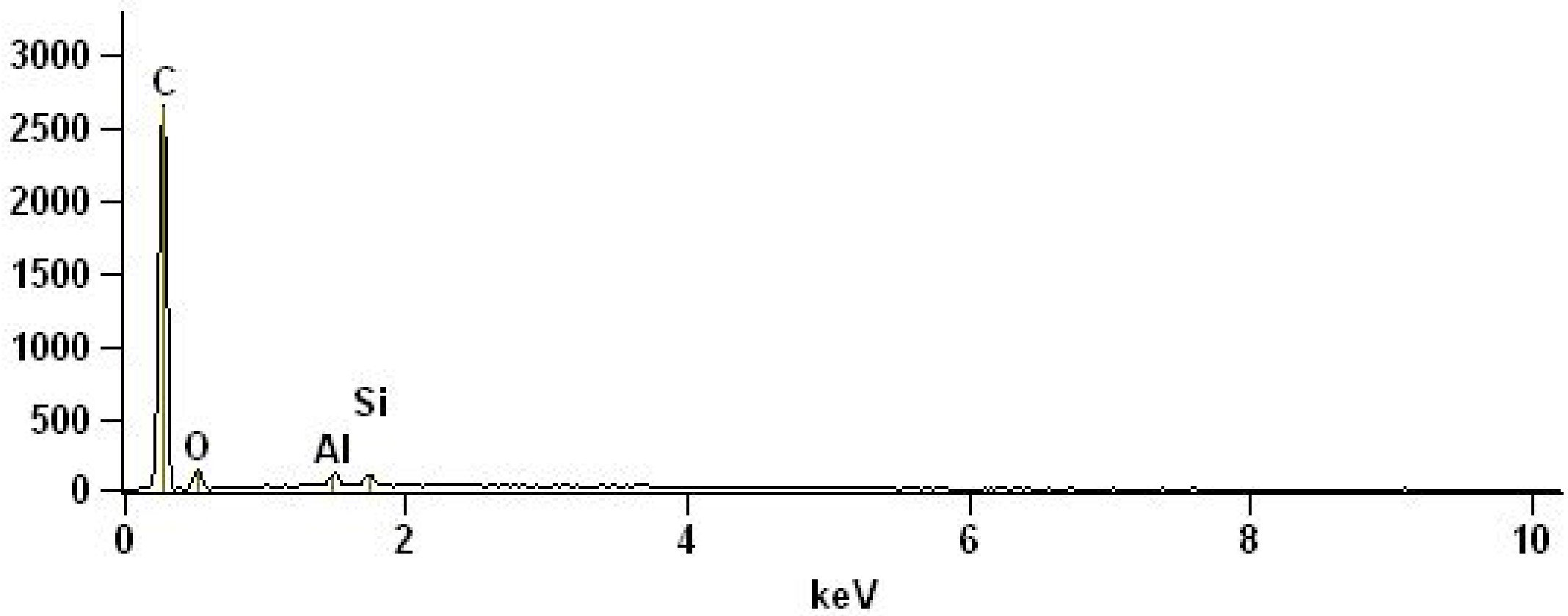

Figure S2. (a) SEM micrographs indicating the selected point analysis regions and the corresponding EDS spectra of the 3D-CB electrode at points 1, 2, and 3 (a1–a3). Accelerating voltage: 15.0 kV.

(a)

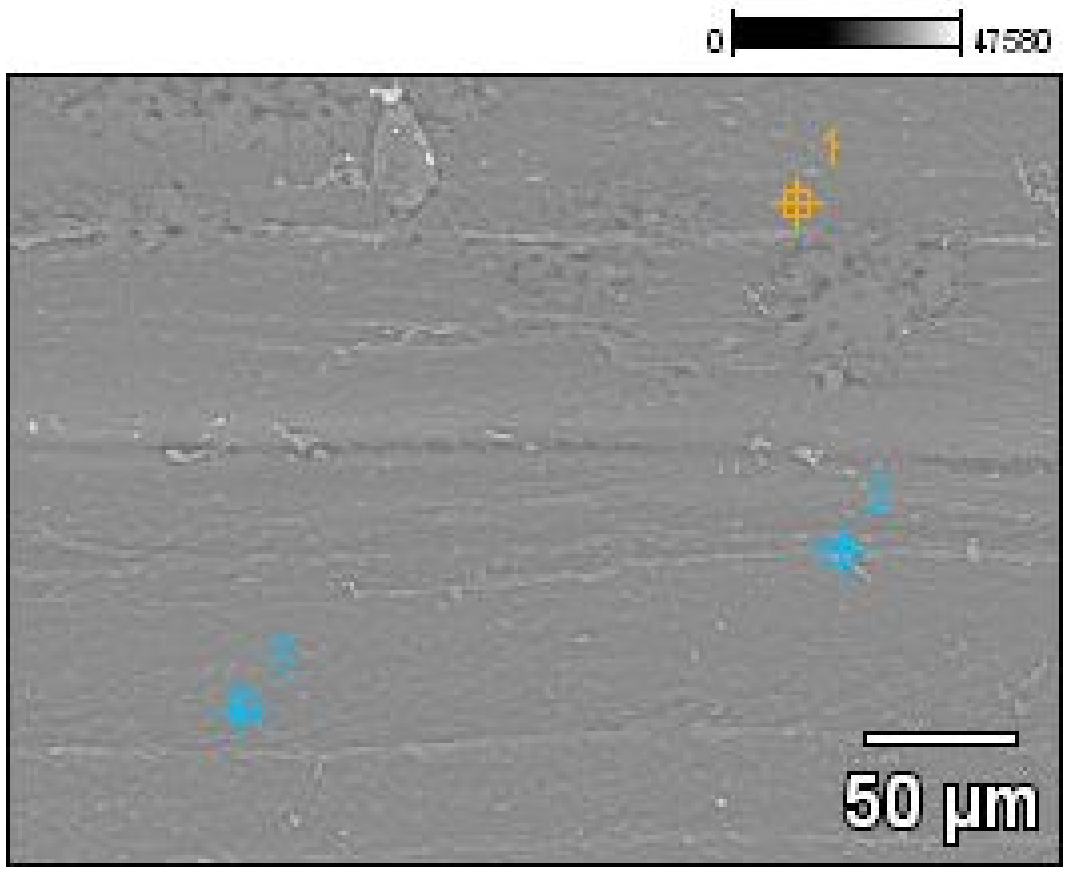
0
47580
1
2
3
50 µm

(a1)

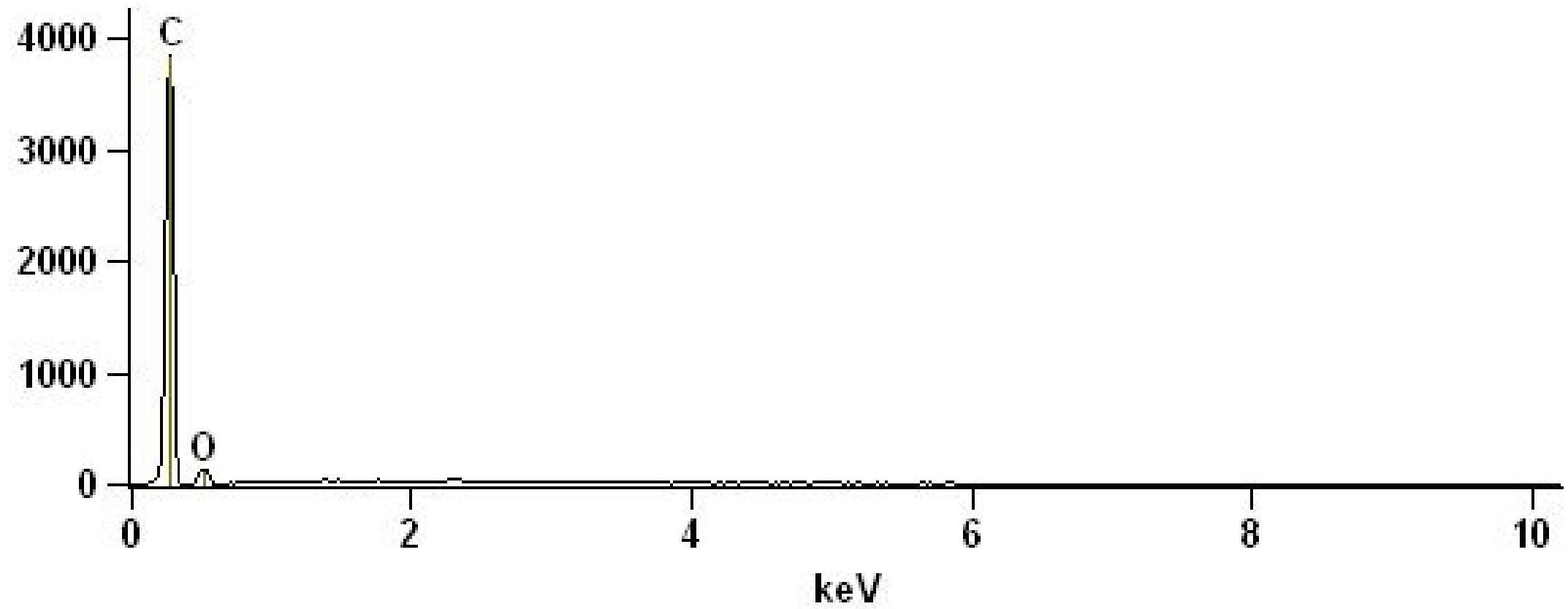
C
O
4000
3000
2000
1000
0
0
2
4
6
8
10
keV

(a2)

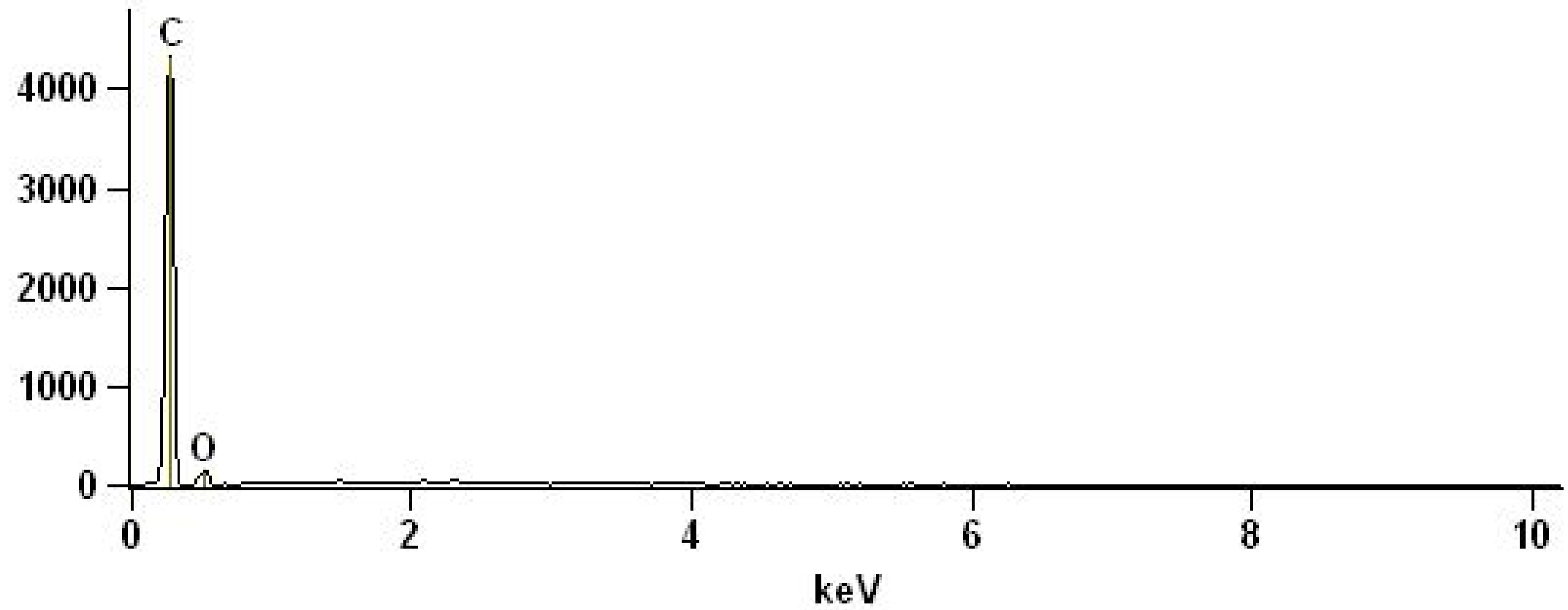
C
O
4000
3000
2000
1000
0
0
2
4
6
8
10
keV

(a3)

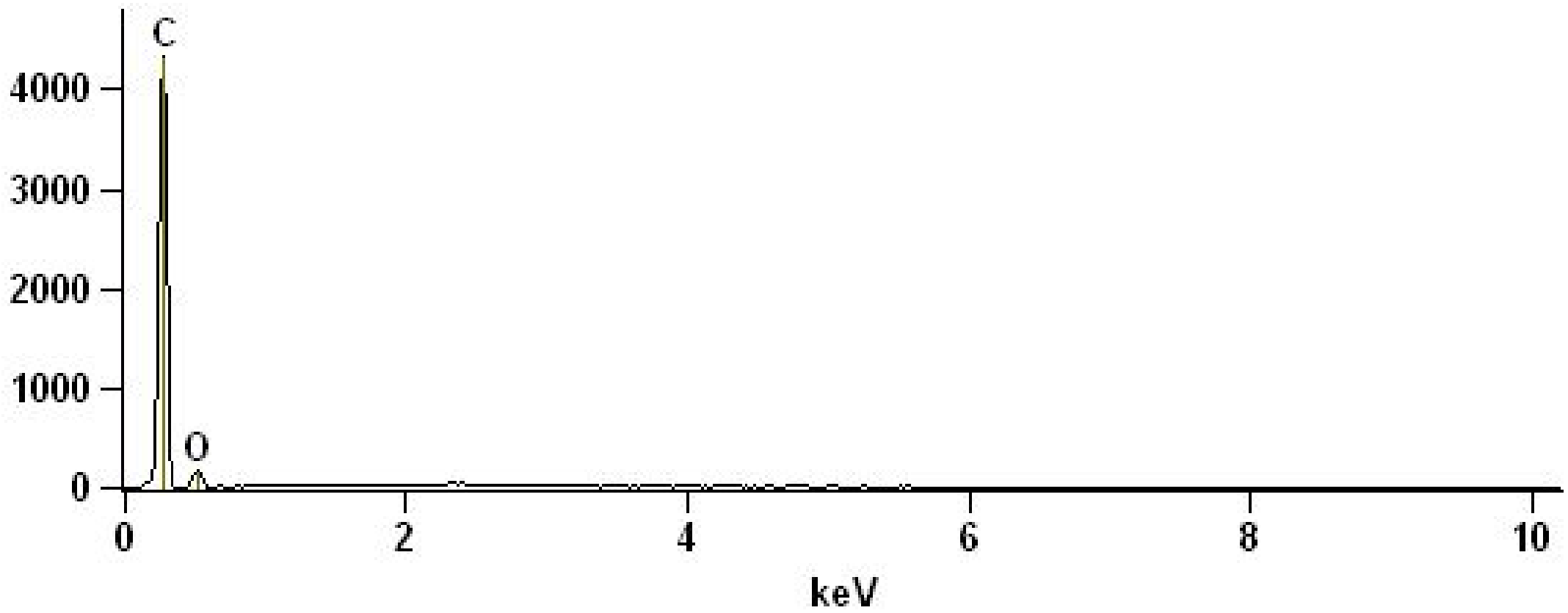

Figure S3. (a) SEM micrographs indicating the selected point analysis regions and the corresponding EDS spectra of the 3D-CB/HEC-CH electrode at points 1, 2, and 3 (a1–a3). Accelerating voltage: 15.0 kV.

(a)

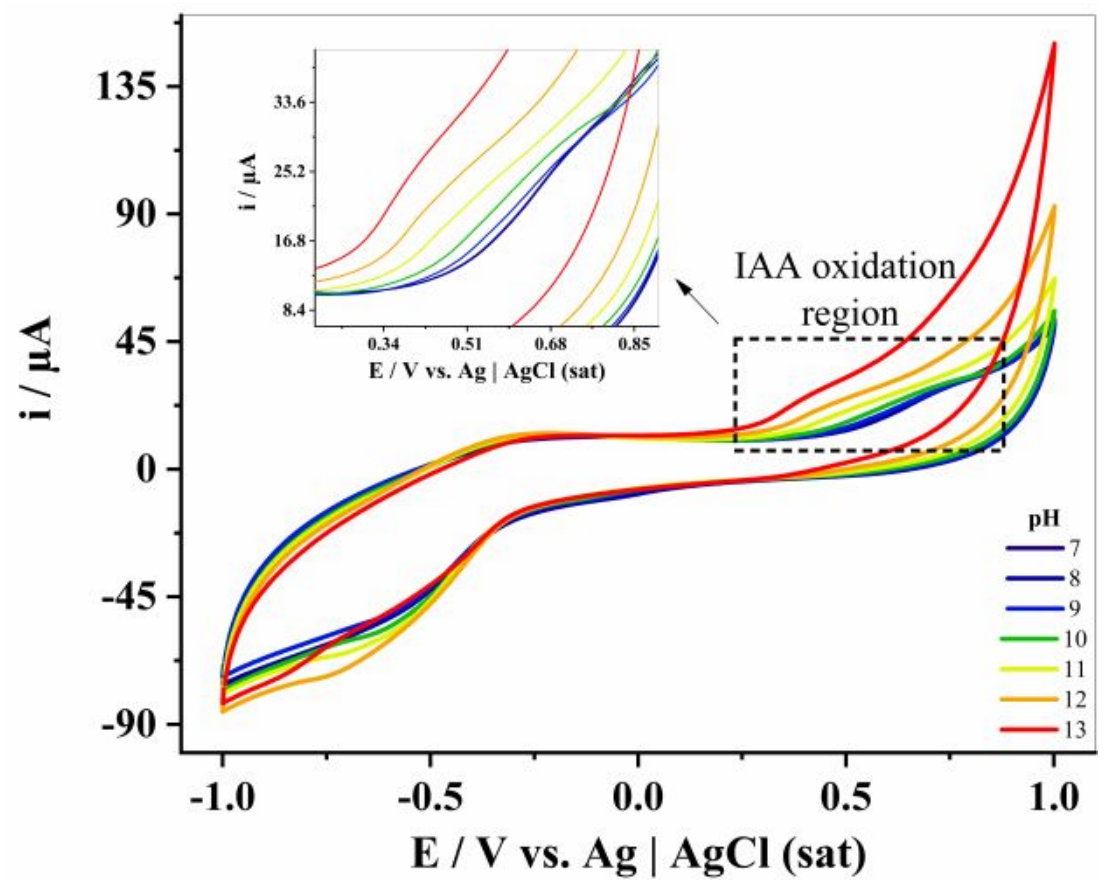

(b)

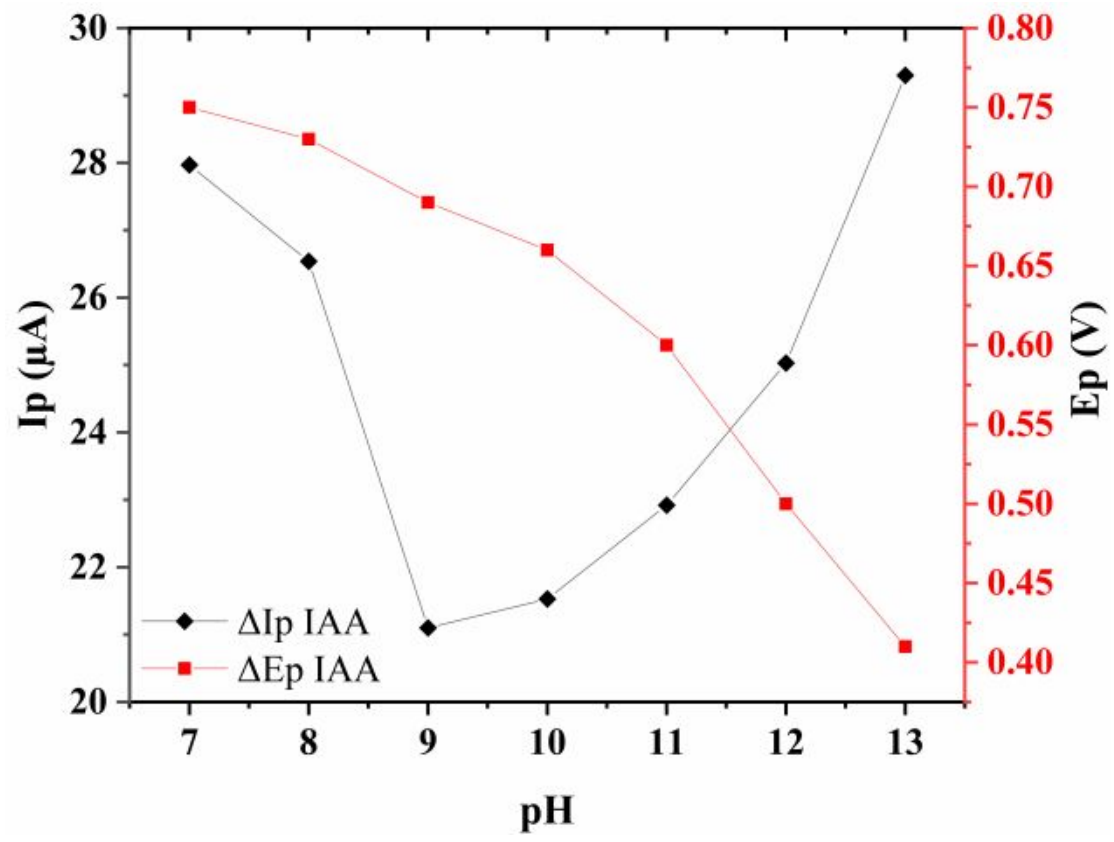

(c)

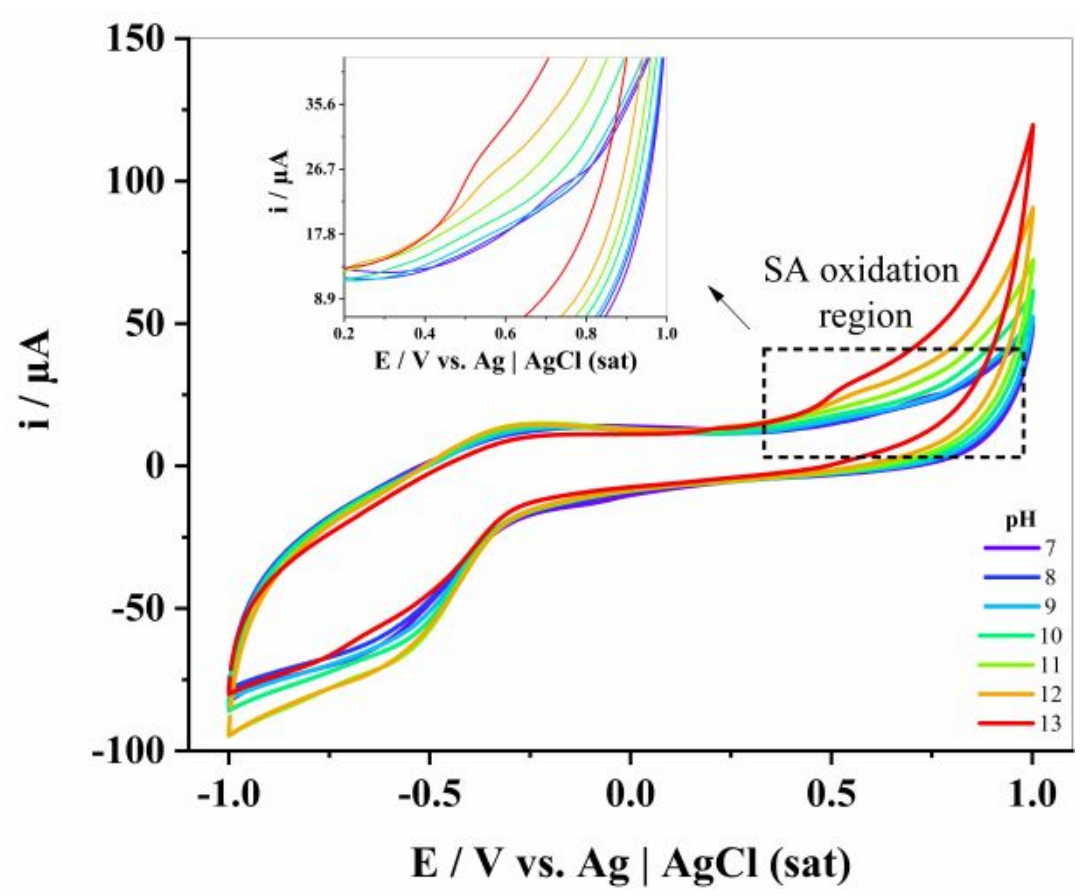

(d)

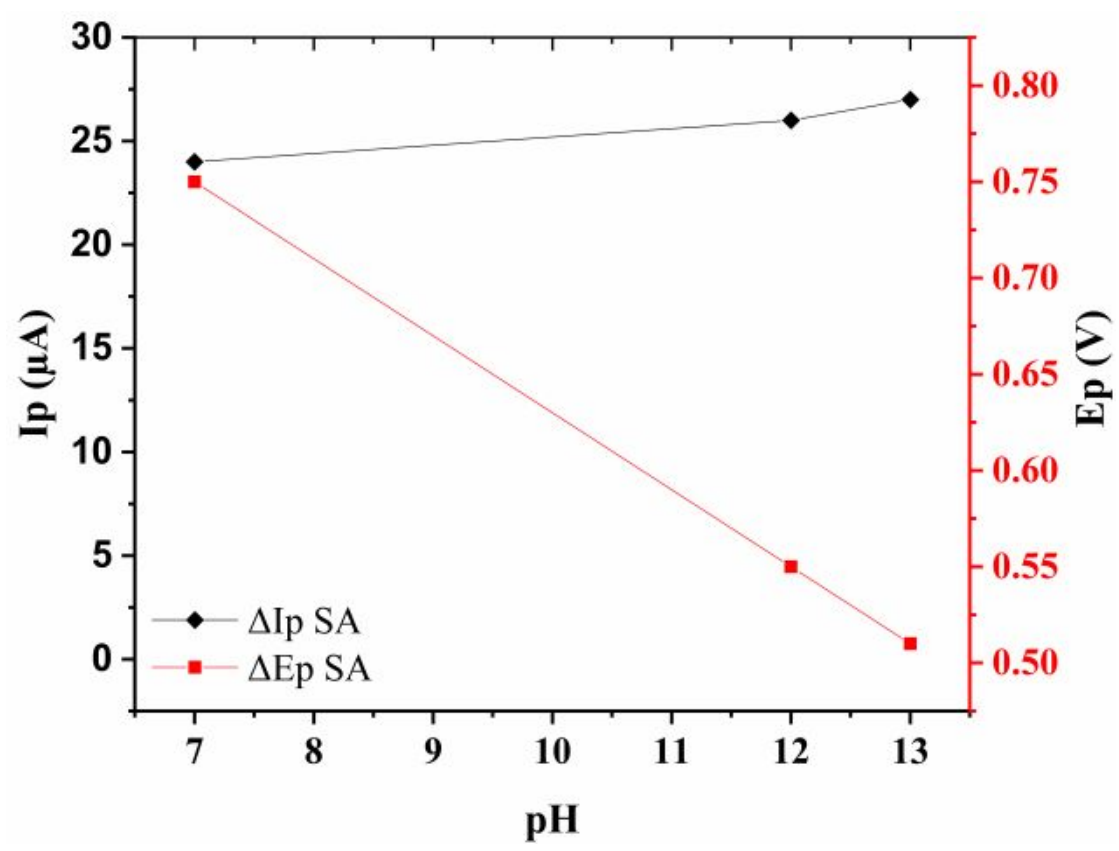

Figure S4. Effect of pH on the electrochemical response of the 3D-CB/HEC–CH electrode for IAA and SA oxidation. (a) and (c) Cyclic voltammograms of IAA and SA (100 μM) recorded in Britton–Robinson buffer at different pH values; (b) and (d) variation of anodic peak current (I_p) and anodic peak potential (E_p) of IAA as a function of pH; (c) cyclic voltammograms of SA (100 μM) recorded in Britton–Robinson buffer at different pH values. Scan rate: 50 mV s^{-1}; step potential: 5 mV; potential window: −1.00 to +1.00 V vs. Ag|AgCl (sat.).

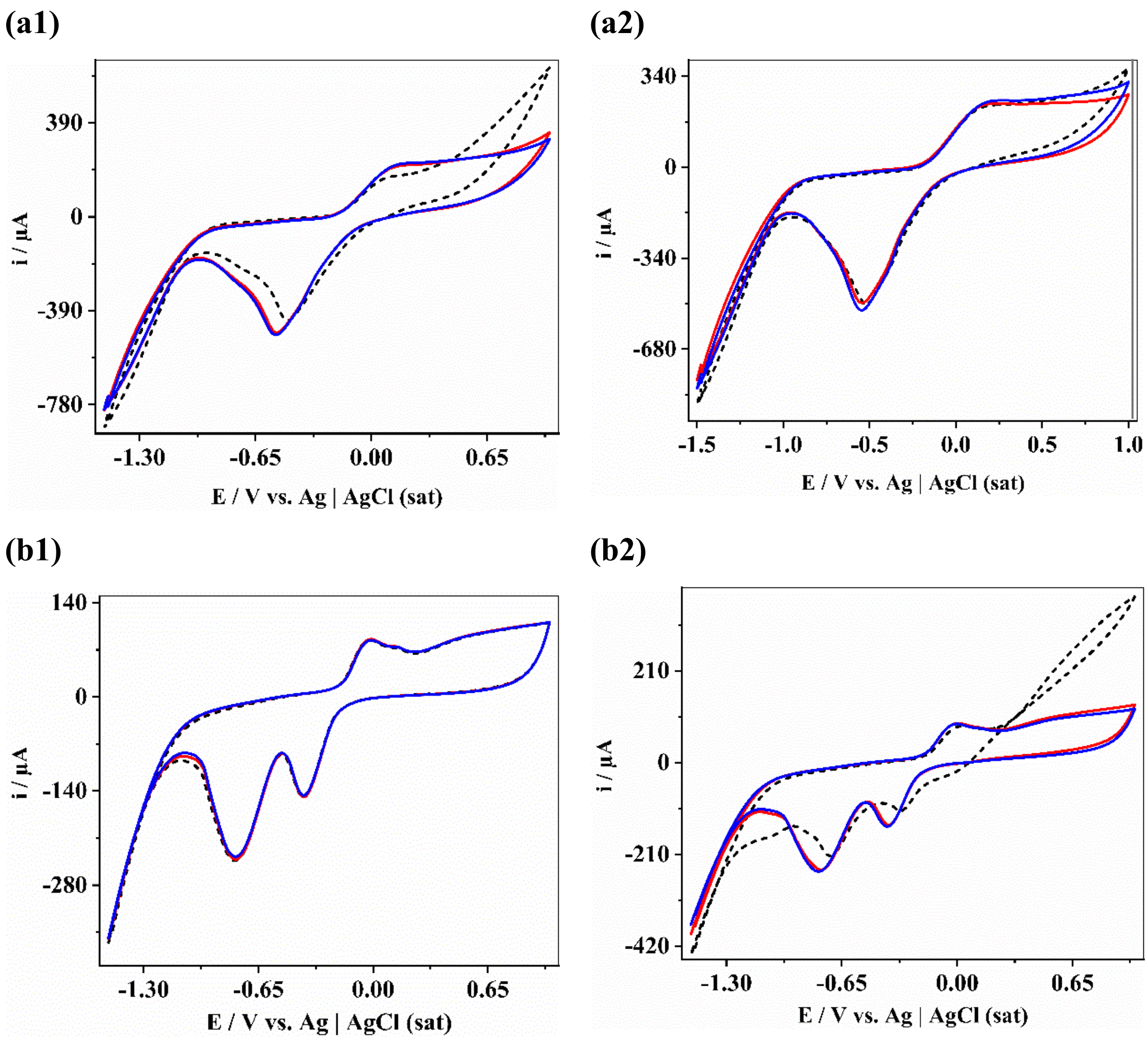

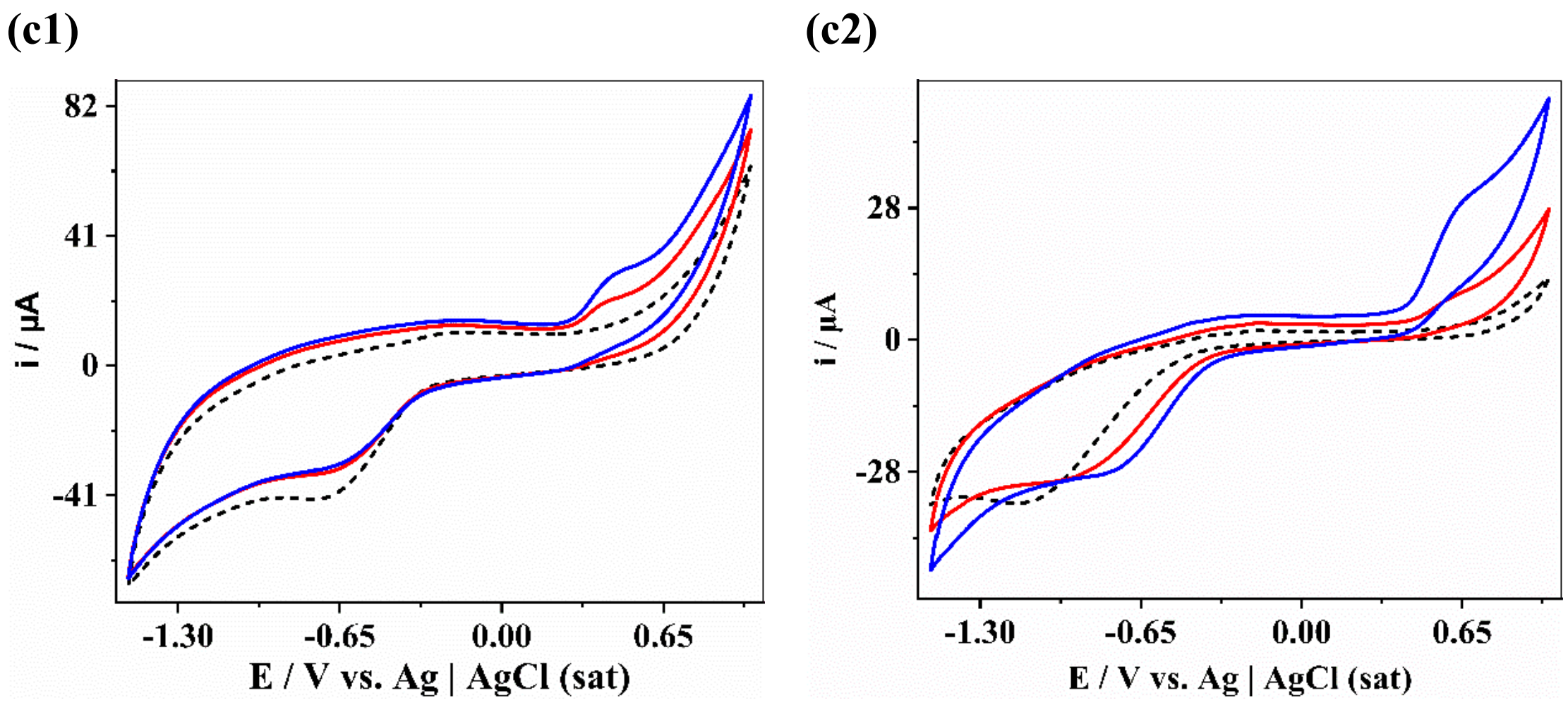

Figure S5. Comparative CV responses of the 3D-CB/HEC–CH electrode in PBS buffer 0.10 M (pH 7) (a), borate buffer 0.10 M (pH 9) (b) and sodium hydroxide solution 0.50 M (pH 13) (c), IAA (1) and SA (2), following the addition of the analytes at 50 µM (red line) and 125 µM (blue line). Scan rate: 50 mV s^{-1}; step potential: 5 mV; potential window: −1.50 to +1.00 V vs. Ag|AgCl (sat.).

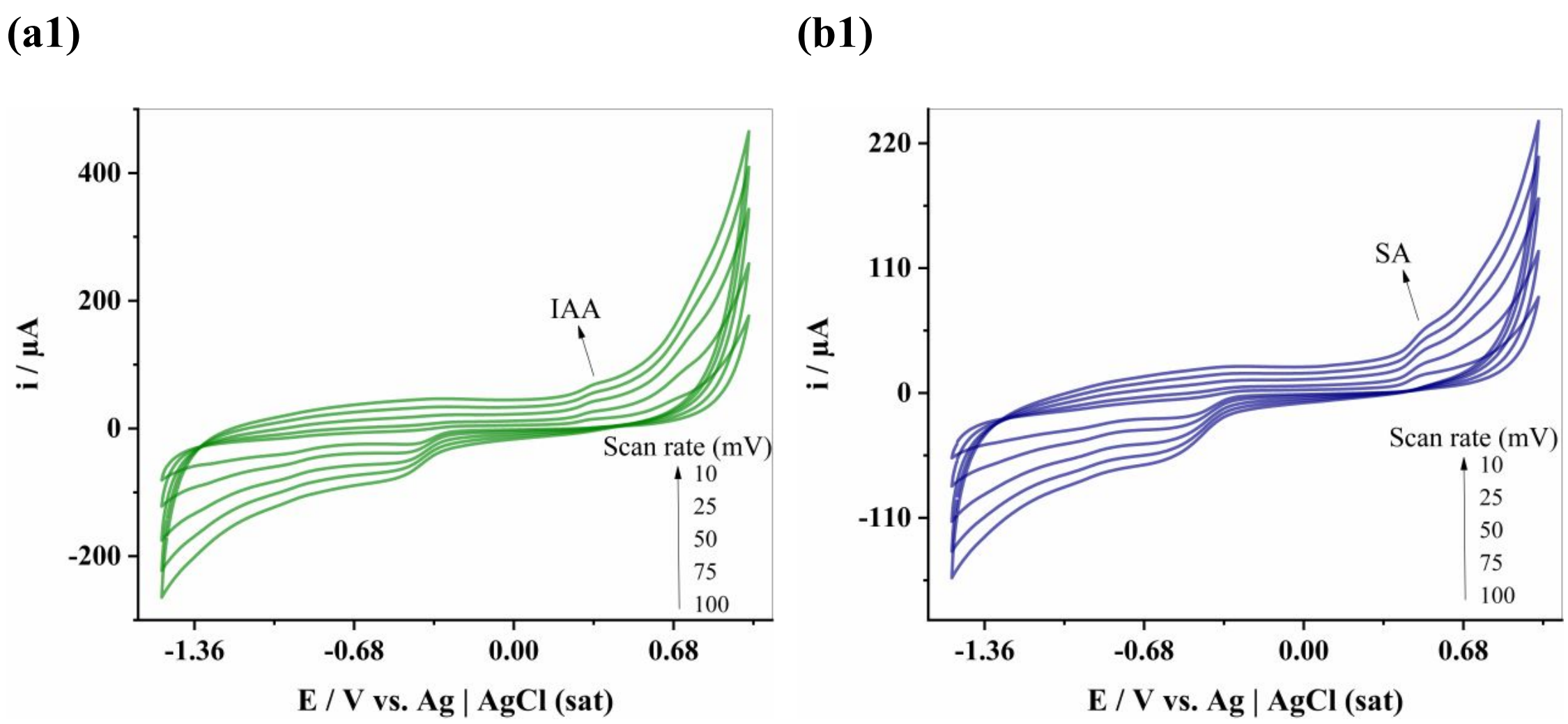

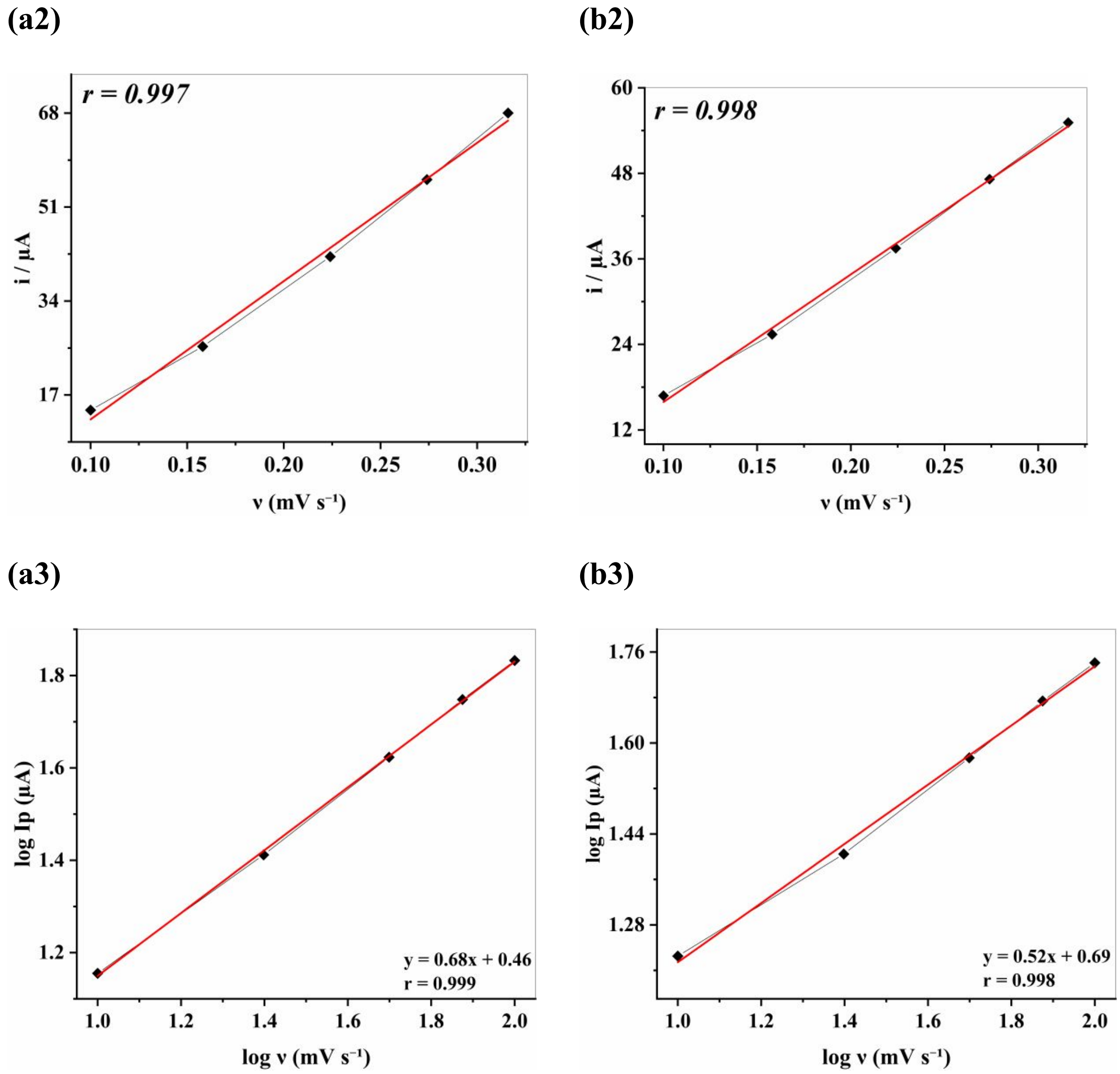

Figure S6. Effect of scan rate on the electrochemical response of the 3D-CB/HEC–CH electrode for IAA and SA oxidation. Cyclic voltammograms recorded at different scan rates for IAA (a1) and SA (b1); linear relationships between anodic peak current (I_p) and the square root of scan rate ($\nu^{1/2}$) for IAA (a2) and SA (b2); and linear relationships between log $I_p$and log ν for IAA (a3) and SA (b3). Experimental conditions: IAA and SA concentration of 450 µM; scan rate range: 10–100 mV s^{-1}; step potential: 5 mV; potential window: −1.50 to +1.00 V vs. Ag|AgCl (sat.).

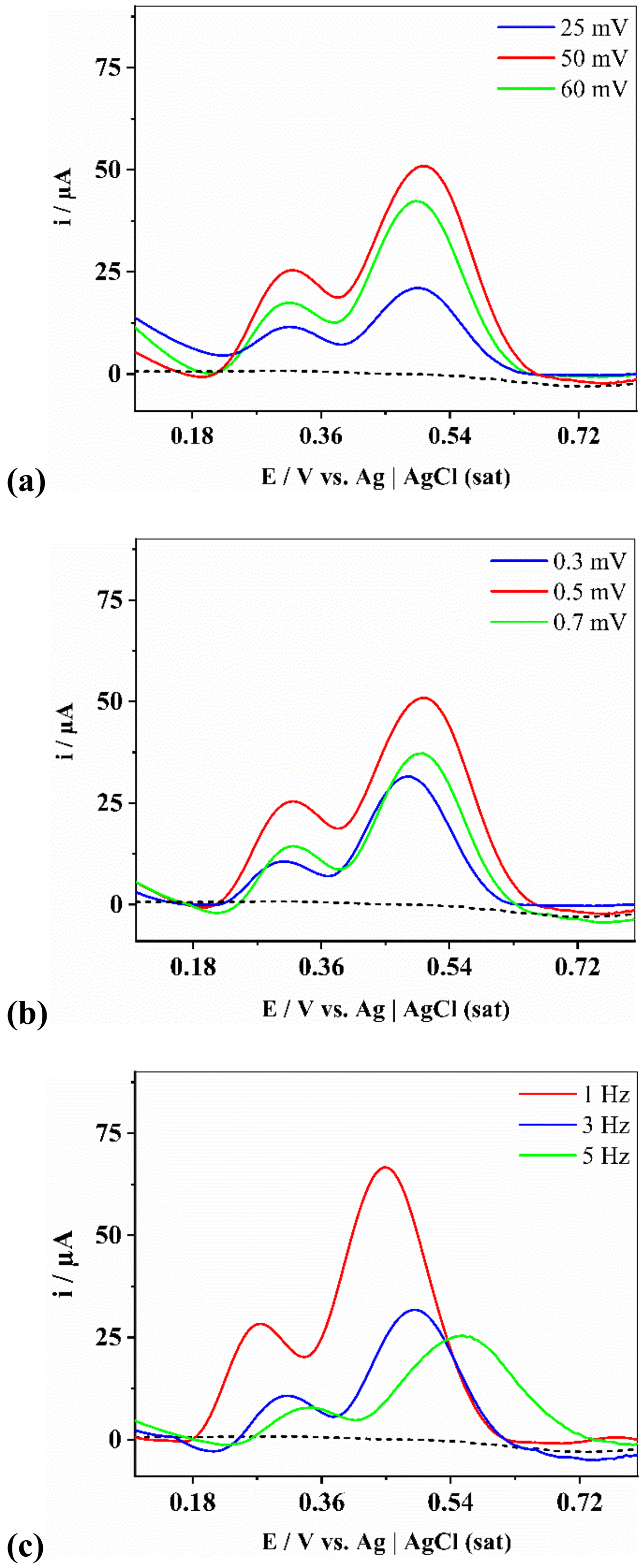

Figure S7. Optimization of the SWV parameters for simultaneous IAA and SA determination. (a) Modulation amplitude. (b) Step potential. (c) Frequency. Other SWV conditions: (a) f = 3 Hz; (ΔE_s) = 0.5 mV. (b) a = 50 mV; f = 3 Hz. (c) a = 50 mV; (ΔE_s) = 0.5 mV. Experimental conditions: [IAA] = 50 µM and [SA] = 50 µM; potential scan from +0.10 to +0.80 V vs. Ag|AgCl (sat.); supporting electrolyte: sodium hydroxide solution (pH 13).

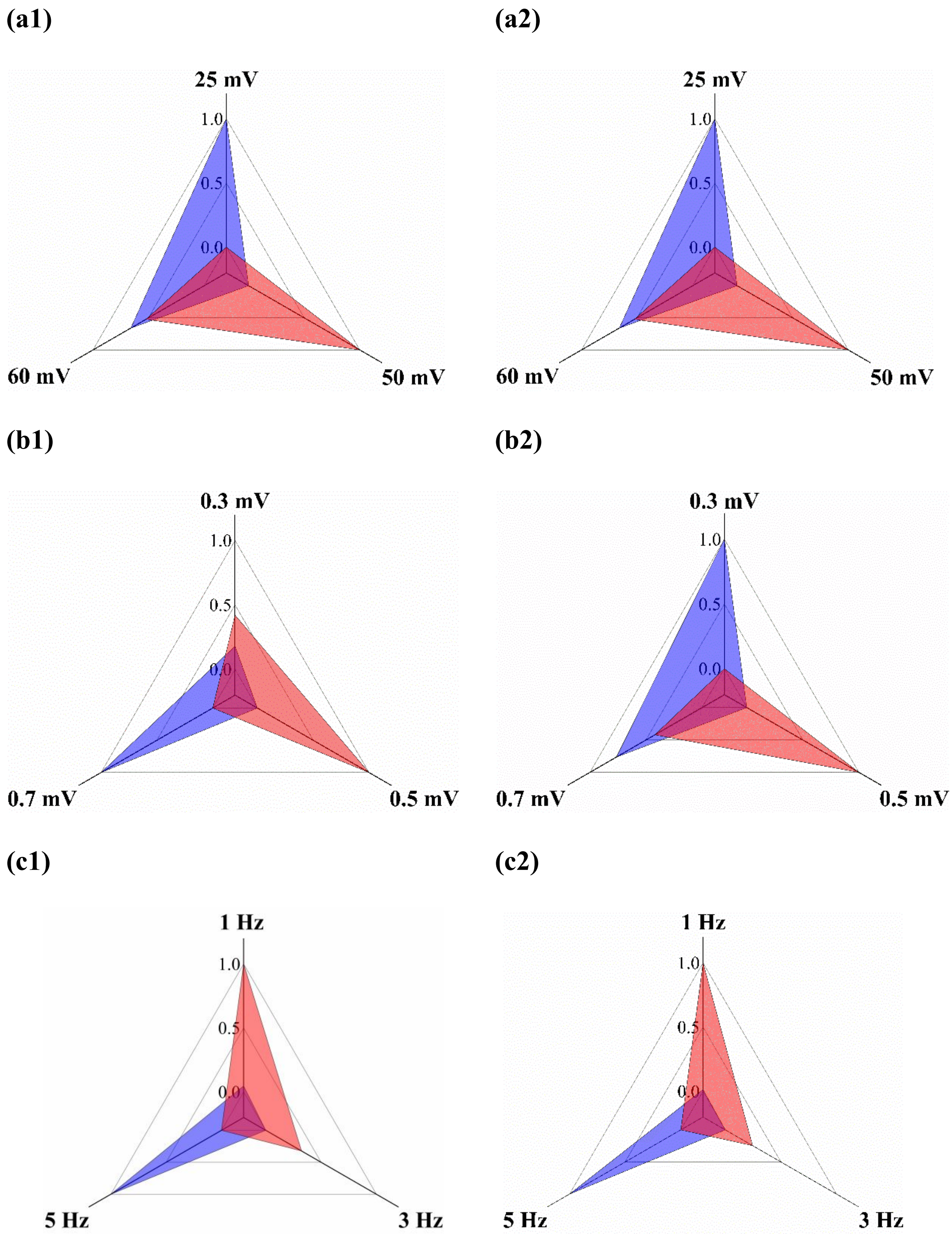

Figure S8. Radar plots used for the optimization of the SWV parameters for the simultaneous determination of IAA and SA. (a) Modulation amplitude, (b) frequency, and (c) step potential. For each parameter, IAA (1) and SA (2) are shown in separate radar plots. Peak-quality metrics were FWHM (▲) and PWHH (▲),

with all data normalized to enable direct visual comparison among the evaluated SWV conditions. Experimental conditions: see Figure S7.

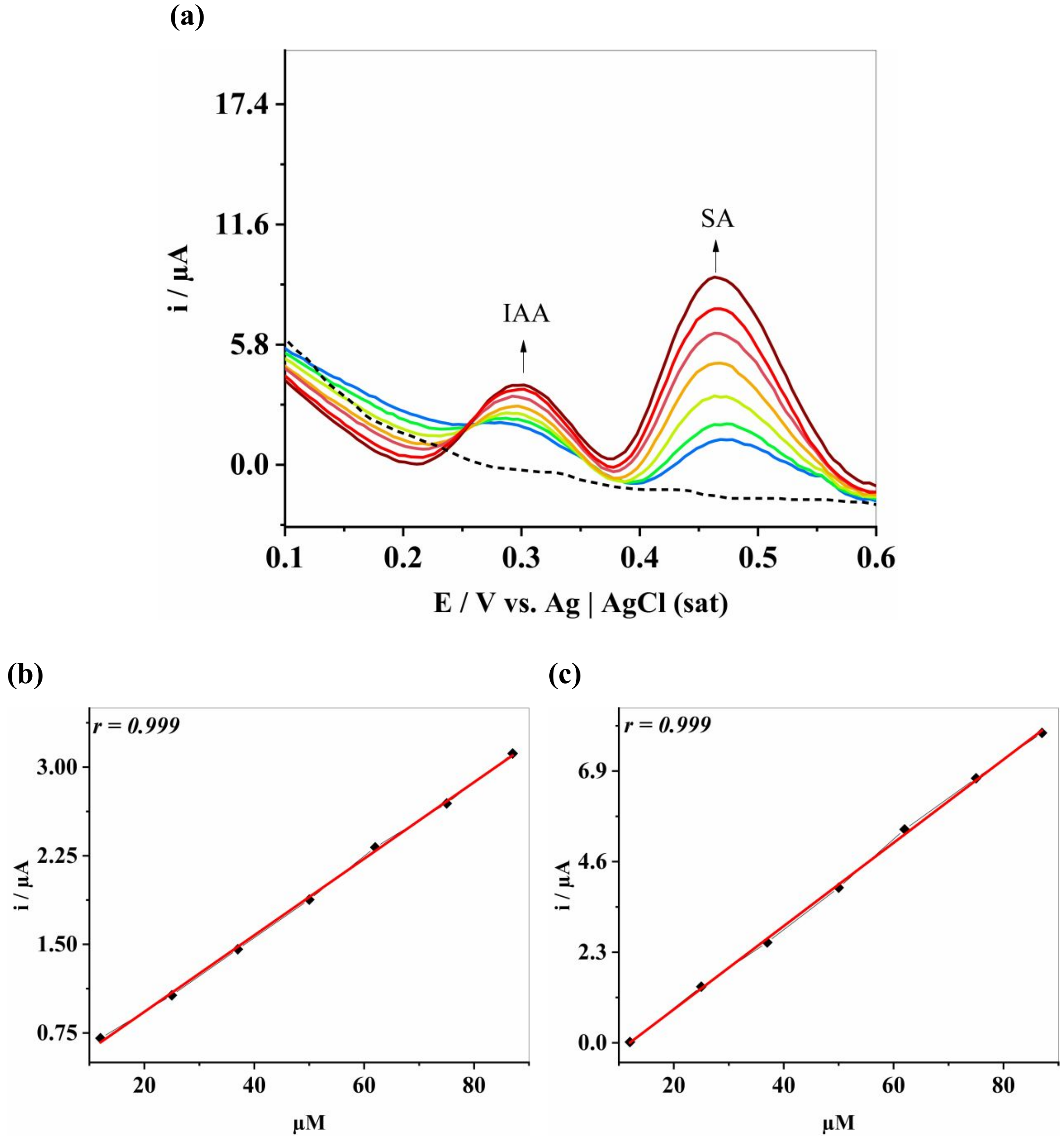

Figure S9. (a) Baseline-corrected SWV responses (n = 3) obtained after injections of standard solutions containing increasing concentrations of IAA and SA (12–87 µM) at the 3D-CB/HEC–CH electrode, using sodium hydroxide solution (pH 13) as the supporting electrolyte (▬ ▬). (b) Calibration curve of IAA (r = 0.999), obtained from the net peak heights. (c) Calibration curve of SA (r = 0.999), obtained under the same conditions as the IAA. SWV parameters: a = 50 mV, f = 1 Hz and (ΔE_s) = 0.5 mV.

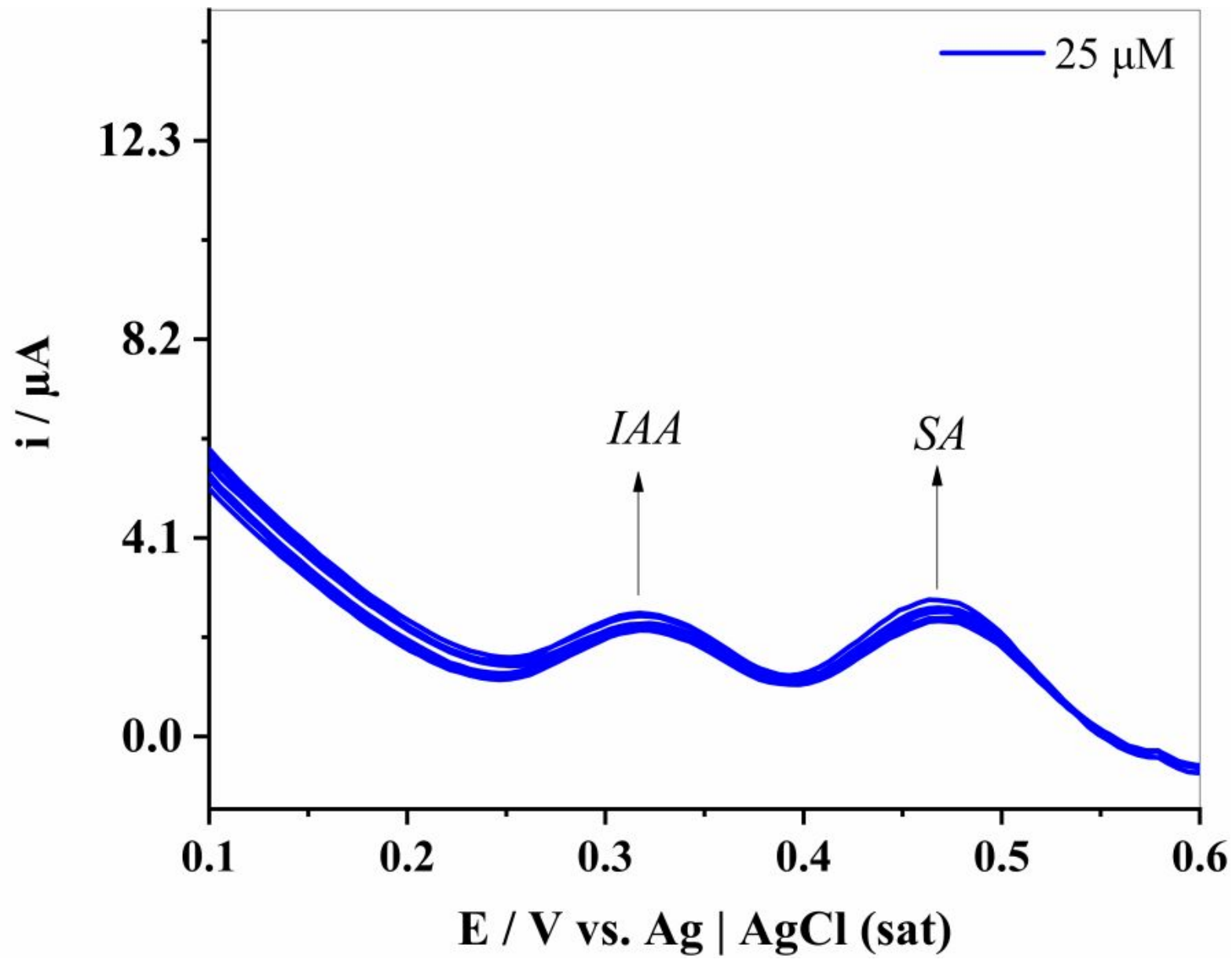

Figure S10. Baseline-corrected SWV responses (n = 3) obtained for a standard solution containing 25 µM IAA and SA at three independently prepared 3D-CB/HEC–CH electrodes, using sodium hydroxide solution (pH 13) as the supporting electrolyte, evaluated as an additional reproducibility parameter to intra- and inter-day precision. RSD values of 4.55% for IAA and 4.59% for SA were obtained across the three electrodes. SWV parameters: see Figure S9.

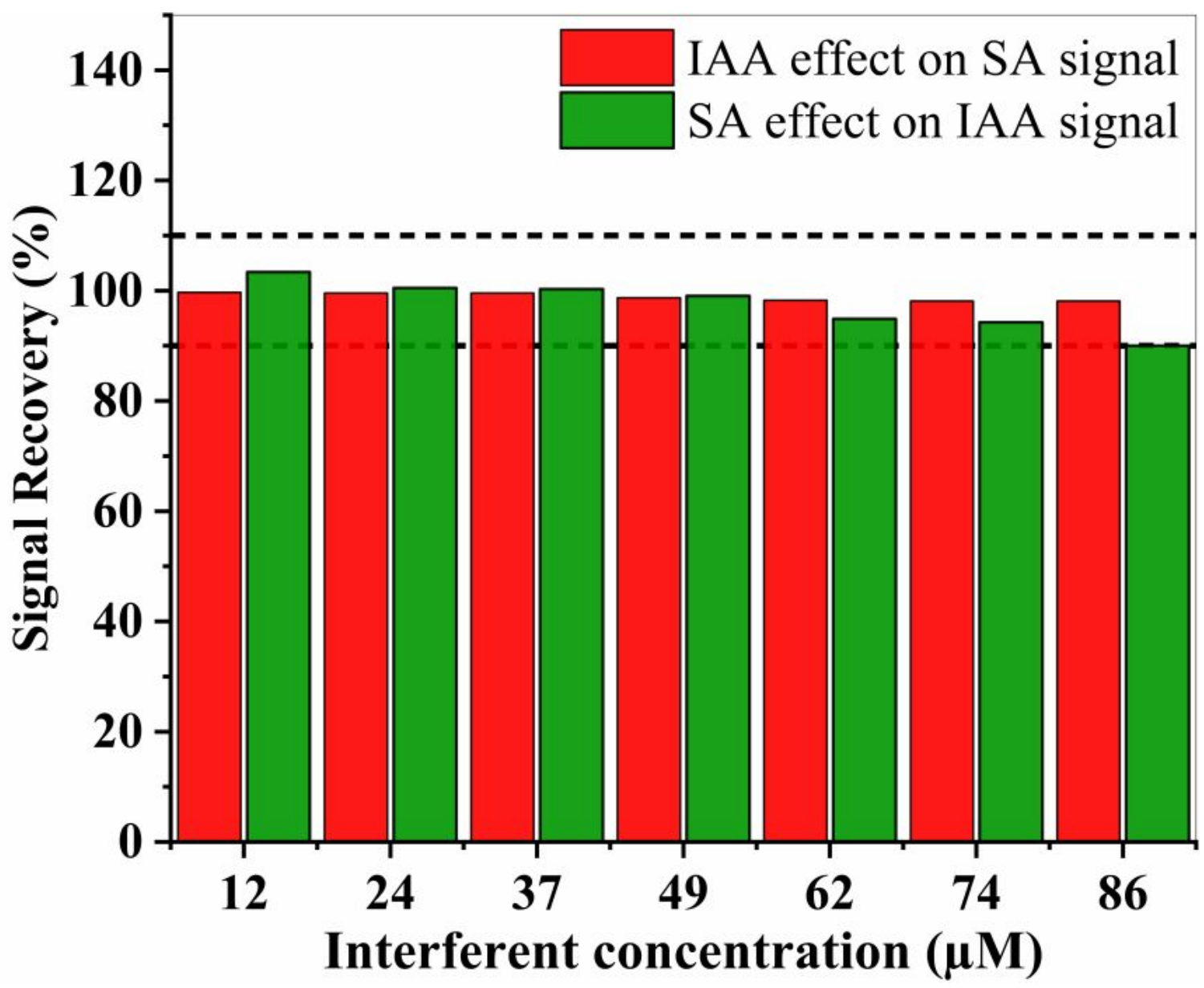

Figure S11. Effect of IAA and SA on each other's electrochemical response within the working range. Red bars (■) represent the SA signal in the presence of increasing IAA concentrations; green bars (■) represent the IAA signal in the presence of increasing SA concentrations. The mutual influence was evaluated by keeping one analyte at a fixed concentration and adding increasing concentrations of the other analyte

within the established working range, up to a 1:1 ratio. The dashed black band marks ±10% (▬ ▬). All values lay within this range. Experimental conditions: potential scan from +0.10 to +0.80 V vs. Ag|AgCl (sat.); supporting electrolyte: sodium hydroxide solution (pH 13). SWV parameters: see Figure S9.

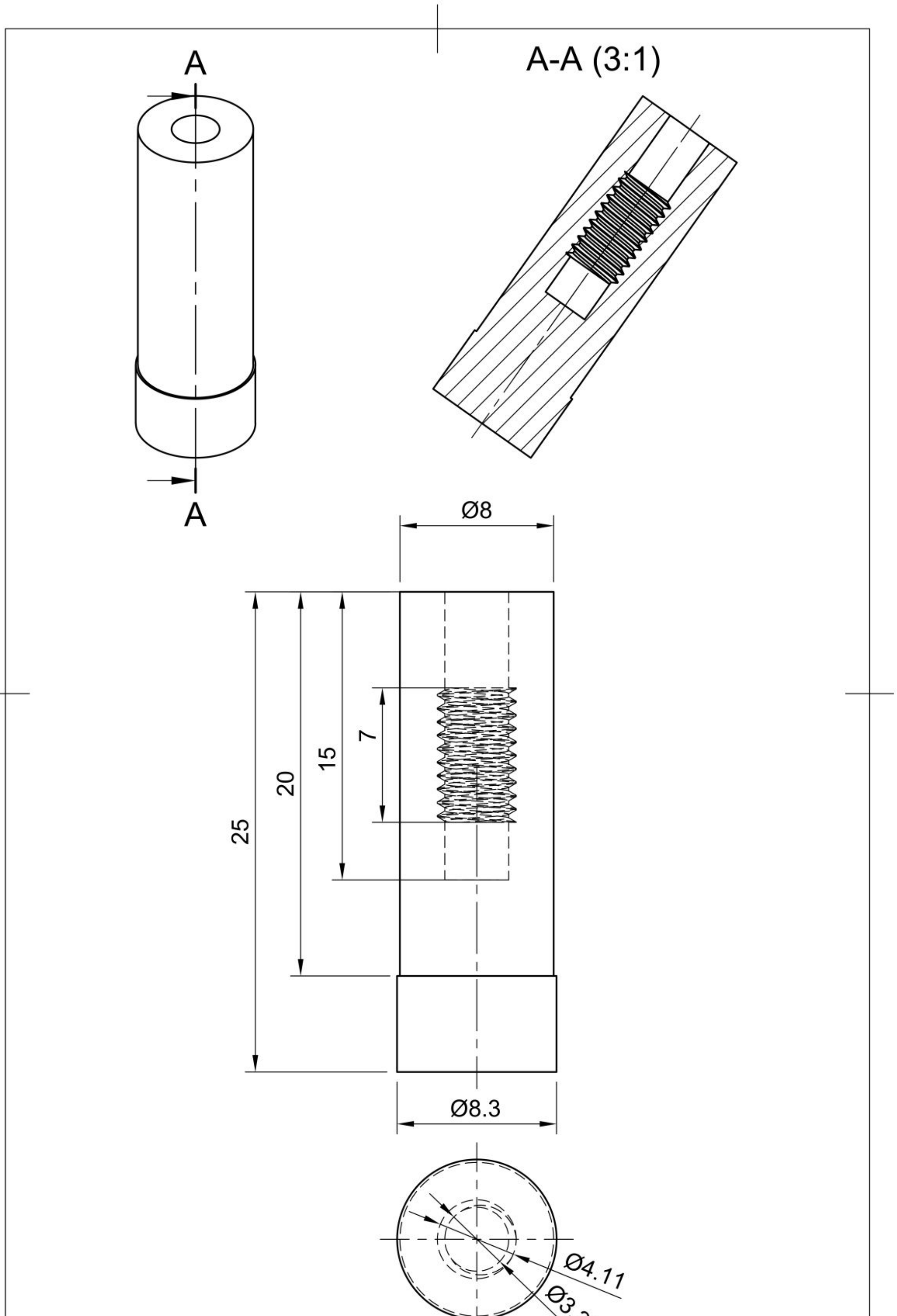

Figure S12. STL design file of the 3D-printed carbon black (3D-CB) electrode used as the working electrode substrate, prepared in Autodesk Fusion and sliced in Creality Slicer prior to printing on a Creality Ender 2 Pro printer, provided to ensure full reproducibility of the electrode fabrication.

	IAA Peak Height (µA)				SA Peak Height (µA)			
**Concentration (µM)**	**R1**	**R2**	**R3**	**Mean (µA)**	**R1**	**R2**	**R3**	**Mean (µA)**
**12**	0.704	0.708	0.706	0.705	0.019	0.019	0.019	0.019
**25**	1.072	1.068	1.070	1.068	1.420	1.411	1.433	1.424
**37**	1.457	1.462	1.461	1.458	2.550	2.563	2.542	2.548
**50**	1.884	1.876	1.880	1.878	3.934	3.950	3.941	3.937
**62**	2.323	2.323	2.318	2.321	5.423	5.432	5.413	5.418
**75**	2.690	2.685	2.695	2.692	6.711	6.734	6.722	6.718
**87**	3.123	3.119	3.118	3.115	7.882	7.861	7.873	7.869

Table S1: Baseline-corrected SWV responses (n = 3) for increasing concentrations of IAA and SA (12–87 µM) recorded at the 3D-CB/HEC-CH electrode in sodium hydroxide solution (pH 13). Calibration curves for IAA (Fig. S9b) and SA (Fig. S9c) were constructed from the net peak heights.

	Intra-day precision				Inter-day precision			
**Run**	**IAA - 12 µM**	**IAA - 62 µM**	**SA - 12 µM**	**SA - 62 µM**	**IAA - 12 µM**	**IAA - 62 µM**	**SA - 12 µM**	**SA - 62 µM**
**1**	0.959	5.575	0.410	5.409	1.013	6.047	0.402	5.449
**2**	0.958	5.773	0.418	5.112	0.987	5.960	0.424	5.405
**3**	0.949	5.878	0.409	5.415	0.935	5.754	0.408	5.332
**4**	0.971	5.699	0.420	5.481	0.954	5.812	0.405	5.477
**5**	0.977	5.646	0.417	5.794	0.935	5.805	0.394	5.427
**6**	0.954	5.720	0.417	5.537	0.919	5.732	0.368	5.501
**7**	0.964	5.862	0.364	5.319	0.952	5.730	0.413	5.614
**8**	0.970	5.766	0.422	5.580	0.934	5.755	0.417	5.362
**Mean (µA)**	0.963	5.740	0.410	5.456	0.954	5.823	0.404	5.446

Table S2: Raw SWV data (n = 8 successive measurements per concentration level) used to evaluate the intra- and inter-day precision and response stability of the 3D-CB/HEC-CH electrode for IAA and SA determination; data used to calculate the mean, SD, and RSD (%) values presented in Table 1.

	**R1**	**R2**	**R3**	**Mean (µA)**		**R1**	**R2**	**R3**	**Mean (µA)**
**Fixed analyte: SA**	7.380	7.390	7.394	7.388	**Fixed analyte: IAA**	3.283	3.291	3.299	3.291
**IAA concentration added (µM)**					**SA concentration added (µM)**				
**12**	7.358	7.368	7.372	7.366	**12**	3.494	3.302	3.410	3.602
**25**	7.348	7.356	7.364	7.356	**25**	3.299	3.307	3.315	3.307

**37**	7.349	7.357	7.365	7.357	**37**	3.293	3.301	3.309	3.301
**50**	7.285	7.293	7.301	7.293	**50**	3.252	3.260	3.268	3.260
**62**	7.248	7.257	7.266	7.257	**62**	3.115	3.123	3.131	3.123
**75**	7.240	7.249	7.258	7.249	**75**	3.094	3.102	3.110	2.802
**87**	7.241	7.249	7.257	7.249	**87**	2.954	2.962	2.970	2.762

Table S3: Triplicate SWV measurements used to evaluate the mutual influence of IAA and SA on their electrochemical responses at concentration ratios ranging from progressive additions up to 1:1 and a maximum concentration of 87 μM. Data used to construct the interference plots shown in Figure S11.

	**IAA signal (μA)**				**SA signal (μA)**			
**Interferent**	**R1**	**R2**	**R3**	**Mean (μA)**	**R1**	**R2**	**R3**	**Mean (μA)**
Analyte only	3.060	3.080	3.100	3.079	7.151	7.170	7.183	7.168
Gallic acid	2.980	3.020	3.001	2.996	7.109	7.131	7.144	7.128
Analyte only	2.790	2.810	2.830	2.811	7.231	7.252	7.264	7.249
Ascorbic acid	2.790	2.770	2.810	2.786	7.087	7.102	7.126	7.105
Analyte only	3.120	3.102	3.140	3.119	7.190	7.199	7.214	7.201
Sucrose	3.170	3.230	3.200	3.203	7.241	7.263	7.273	7.259
Analyte only	3.070	3.050	3.090	3.071	7.218	7.236	7.248	7.234
Kinetin	3.013	2.980	2.960	2.977	7.072	7.096	7.111	7.093
Analyte only	3.070	3.050	3.090	3.070	7.500	7.200	7.800	7.500
Catechol	3.240	3.180	3.220	3.213	7.700	7.000	7.500	7.400
Analyte only	3.070	3.050	3.090	3.070	7.500	7.200	7.800	7.500
Indole-3-butyric acid (IBA)	3.140	3.100	3.170	3.137	7.600	7.400	7.700	7.567
Analyte only	3.070	3.050	3.090	3.070	7.500	7.200	7.800	7.500
Abscisic acid (ABA)	3.090	3.070	3.110	3.090	7.300	7.700	7.300	7.433
Analyte only	3.070	3.050	3.090	3.070	7.500	7.200	7.800	7.500
Rutin	3.050	3.090	3.040	3.060	7.400	7.300	7.600	7.433
Analyte only	3.070	3.050	3.090	3.070	7.500	7.200	7.800	7.500
Citric acid	3.020	3.980	3.000	3.000	7.200	7.000	7.300	7.167
Analyte only	3.070	3.050	3.090	3.070	7.500	7.200	7.800	7.500
L-Phenylalanine (Phe)	3.130	3.160	3.120	3.137	7.800	7.600	7.900	7.767
Analyte only	3.070	3.050	3.090	3.070	7.500	7.200	7.800	7.500
Benzoic acid	3.010	3.980	3.030	3.010	7.300	7.100	7.400	7.267

Table S4: Triplicate SWV measurements used to evaluate the effect of possible interferents on the electrochemical responses of IAA and SA (87 µM) at a 1:1 analyte/interferent ratio in sodium hydroxide solution (pH 13). Data used to construct the interference plots shown in Figure 6.

	**IAA signal (µA)**				**SA signal (µA)**			
**Condition**	**R1**	**R2**	**R3**	**Mean (µA)**	**R1**	**R2**	**R3**	**Mean (µA)**
Nominal conditions	1.646	1.639	1.483	1.589	2.914	2.847	2.839	2.867
20 ºC	1.596	1.539	1.493	1.543	2.954	2.877	2.829	2.887
Operator 2	1.563	1.550	1.542	1.552	2.982	2.825	2.851	2.886
0.45 M electrolyte	1.505	1.565	1.508	1.526	2.913	2.842	2.869	2.875
0.55 M electrolyte	1.528	1.694	1.473	1.565	2.805	2.950	2.875	2.877

Table S5: Raw data (n = 3) obtained for the robustness evaluation of the 3D-CB/HEC-CH electrode by varying the temperature, operator, and supporting-electrolyte concentration while maintaining all other experimental conditions constant.

	**IAA signal (µA)**				**SA signal (µA)**			
**Matrix**	**Soybean**				**Soybean**			
**Spike (µM)**	**R1**	**R2**	**R3**	**Mean (µA)**	**R1**	**R2**	**R3**	**Mean (µA)**
**12**	0.523	0.512	0.537	0.524	0.851	0.659	0.792	0.767
**25**	0.827	0.853	0.757	0.812	1.329	1.459	1.190	1.326
**37**	1.022	1.058	1.019	1.033	1.929	1.988	2.174	2.030
**50**	1.362	1.296	1.361	1.340	2.469	2.813	2.565	2.616
**62**	1.626	1.655	1.639	1.640	3.223	3.541	3.384	3.383
**75**	1.967	1.981	1.896	1.948	4.076	3.836	4.072	3.994
**87**	2.282	2.253	2.228	2.254	4.527	4.718	4.675	4.640
**Matrix**	**Maize**				**Maize**			
**Spike (µM)**	**R1**	**R2**	**R3**	**Mean (µA)**	**R1**	**R2**	**R3**	**Mean (µA)**
**12**	0.553	0.477	0.408	0.480	0.904	0.901	0.931	0.913
**25**	0.740	0.771	0.737	0.750	1.340	1.636	1.528	1.502
**37**	0.997	0.992	0.997	0.996	2.162	2.027	2.130	2.107
**50**	1.188	1.233	1.299	1.240	2.610	2.756	2.813	2.726
**62**	1.539	1.518	1.465	1.508	3.360	3.426	3.267	3.351
**75**	1.709	1.840	1.773	1.775	3.881	4.057	3.859	3.933
**87**	1.945	1.892	2.102	1.980	4.486	4.542	4.536	4.522
**Matrix**	**Strawberry**				**Strawberry**			
**Spike (µM)**	**R1**	**R2**	**R3**	**Mean (µA)**	**R1**	**R2**	**R3**	**Mean (µA)**
**12**	2.301	2.000	2.430	2.244	4.025	4.097	4.161	4.094
**25**	2.863	2.857	2.937	2.886	6.251	6.025	5.997	6.091
**37**	3.304	3.567	3.440	3.437	8.122	7.724	7.722	7.856

**50**	4.206	3.802	3.937	3.982	9.518	9.601	9.725	9.615
**62**	4.667	4.215	4.338	4.407	11.180	11.150	10.670	11.000
**75**	5.377	4.761	4.862	5.000	13.060	13.190	13.020	13.090
**87**	5.523	5.645	5.272	5.480	14.850	14.780	14.690	14.770

Table S6: Raw data (n = 3) obtained for the baseline-corrected SWV responses (n = 3) obtained in plants extracts using the standard-addition method under optimized conditions. Calibration curves for IAA and SA were constructed from net peak heights for soybean (Fig.7b–c), maize (Fig. 7e–f), and strawberry (Fig. 7h–i) extracts.

	**IAA signal (µA)**				**SA signal (µA)**			
**Matrix**	**Soybean**				**Soybean**			
**Concentration (µM)**	**R1**	**R2**	**R3**	**Mean (µA)**	**R1**	**R2**	**R3**	**Mean (µA)**
**Real sample**	0.250	0.255	0.251	0.252	0.250	0.243	0.267	0.258
25	0.827	0.853	0.757	0.813	1.382	1.459	1.402	1.414
**75**	1.967	1.981	1.896	1.948	4.076	3.836	4.072	3.994
**Matrix**	**Maize**				**Maize**			
**Concentration (µM)**	**R1**	**R2**	**R3**	**Mean (µA)**	**R1**	**R2**	**R3**	**Mean (µA)**
**Real sample**	0.189	0.212	0.174	0.192	0.349	0.212	0.226	0.262
**25**	0.758	0.714	0.777	0.769	1.584	1.636	1.528	1.583
**75**	1.709	1.840	1.773	1.775	3.881	4.057	3.859	3.933
**Matrix**	**Strawberry**				**Strawberry**			
**Concentration (µM)**	**R1**	**R2**	**R3**	**Mean (µA)**	**R1**	**R2**	**R3**	**Mean (µA)**
**Real sample**	2.268	2.290	2.391	2.316	2.691	2.462	2.631	2.595
**25**	3.331	3.368	3.405	3.368	6.251	6.025	5.997	6.091
**75**	5.523	5.645	5.272	5.480	13.060	13.190	13.020	13.090

Table S7: Concentrations (n = 3) of IAA and SA determined from the standard addition method in plant extract samples used for recovery calculations. These data were employed to obtain the final Recovery (%) values reported in the main manuscript (Table 4). Recovery was calculated as the ratio between the experimentally found concentration and the theoretical added concentration for each spike level under optimized SWV conditions.
